# Single-Cell Analysis of the Plasmablast Response to *Vibrio cholerae* Demonstrates Expansion of Cross-Reactive Memory B Cells

**DOI:** 10.1128/mBio.02021-16

**Published:** 2016-12-20

**Authors:** Robert C. Kauffman, Taufiqur R. Bhuiyan, Rie Nakajima, Leslie M. Mayo-Smith, Rasheduzzaman Rashu, Mohammad Rubel Hoq, Fahima Chowdhury, Ashraful Islam Khan, Atiqur Rahman, Siddhartha K. Bhaumik, Levelle Harris, Justin T. O'Neal, Jessica F. Trost, Nur Haq Alam, Algis Jasinskas, Emmanuel Dotsey, Meagan Kelly, Richelle C. Charles, Peng Xu, Pavol Kováč, Stephen B. Calderwood, Edward T. Ryan, Phillip L. Felgner, Firdausi Qadri, Jens Wrammert, Jason B. Harris

**Affiliations:** a Division of Infectious Disease, Department of Pediatrics, Emory University, School of Medicine, Atlanta, Georgia, USA; b Emory Vaccine Center, Emory University School of Medicine, Atlanta, Georgia, USA; c Infectious Diseases Division, International Centre for Diarrhoeal Disease Research, Bangladesh (icddr,b), Dhaka, Bangladesh; d Department of Medicine, University of California, Irvine, California, USA; e Division of Infectious Diseases, Massachusetts General Hospital, Boston, Massachusetts, USA; f Department of Medicine, Harvard Medical School, Boston, Massachusetts, USA; g National Institute of Diabetes and Digestive and Kidney Diseases, Laboratory of Bioorganic Chemistry, National Institutes of Health, Bethesda, Maryland, USA; h Department of Immunology and Infectious Diseases, Harvard T. H. Chan School of Public Health, Boston, Massachusetts, USA; i Department of Pediatrics, Harvard Medical School, Boston, Massachusetts, USA

## Abstract

We characterized the acute B cell response in adults with cholera by analyzing the repertoire, specificity, and functional characteristics of 138 monoclonal antibodies (MAbs) generated from single-cell-sorted plasmablasts. We found that the cholera-induced responses were characterized by high levels of somatic hypermutation and large clonal expansions. A majority of the expansions targeted cholera toxin (CT) or lipopolysaccharide (LPS). Using a novel proteomics approach, we were able to identify sialidase as another major antigen targeted by the antibody response to *Vibrio cholerae* infection. Antitoxin MAbs targeted both the A and B subunits, and most were also potent neutralizers of enterotoxigenic *Escherichia coli* heat-labile toxin. LPS-specific MAbs uniformly targeted the O-specific polysaccharide, with no detectable responses to either the core or the lipid moiety of LPS. Interestingly, the LPS-specific antibodies varied widely in serotype specificity and functional characteristics. One participant infected with the Ogawa serotype produced highly mutated LPS-specific antibodies that preferentially bound the previously circulating Inaba serotype. This demonstrates durable memory against a polysaccharide antigen presented at the mucosal surface and provides a mechanism for the long-term, partial heterotypic immunity seen following cholera.

## IMPORTANCE

Cholera is a diarrheal disease that results in significant mortality. While oral cholera vaccines are beneficial, they do not achieve equivalent protection compared to infection with *Vibrio cholerae*. Although antibodies likely mediate protection, the mechanisms of immunity following cholera are poorly understood, and a detailed understanding of antibody responses to cholera is of significance for human health. In this study, we characterized the human response to cholera at the single-plasmablast, monoclonal antibody level. Although this approach has not been widely applied to the study of human bacterial infection, we were able to uncover the basis of cross-reactivity between different *V. cholerae* serotypes and the likely impact of prior enterotoxigenic *Escherichia coli* exposure on the response to cholera, as well as identify novel antigenic targets. In addition to improving our understanding of the repertoire and function of the antibody response to cholera in humans, this study has implications for future cholera vaccination efforts.

## INTRODUCTION

*Vibrio cholerae* causes cholera, a severe secretory diarrheal illness. Approximately 2.9 million people develop cholera annually, resulting in an estimated 95,000 deaths (1). *V. cholerae* stably persists in aquatic environments, and over 200 serogroups have been identified. However, the vast majority of cholera cases are caused by the O1 serogroup, which is further subdivided into two serotypes, Inaba and Ogawa. These serotypes differ by the presence or absence of a single 2-O-methyl group in the terminal sugar at the end of the lipopolysaccharide (LPS) O-specific polysaccharide (OSP) (2). Cholera-causing strains of *V. cholerae* colonize the surface of the small intestine, where they produce cholera toxin (CT), an AB5 toxin. The CtxB subunit pentamer binds GM1 gangliosides on the cell surface, leading to endocytosis and cleavage of the CtxA subunit which traffics into the cell. This results in activation of adenylate cyclase, causing secretory diarrhea (3).

Human challenge and longitudinal studies in areas of endemicity demonstrate that an episode of cholera protects against subsequent infection (4, 5). Models suggest that acquired immunity to cholera begins to wane around 5 years after exposure and declines to baseline levels at approximately 10 years following infection (6). The serum vibriocidal antibody is the best-established correlate of protection against cholera (7). Vibriocidal seroconversion following vaccination with an attenuated cholera vaccine is associated with protection against infection (7), and increasing vibriocidal titers are associated with protection against cholera in areas of endemicity (8, 9). However, vibriocidal antibodies decline to preinfection levels before protection wanes (5, 10, 11), suggesting that the vibriocidal antibody response is a marker for other responses that mediate immunity at the mucosal surface. Similarly, circulating levels of CT-specific antibodies are independently associated with protection but only remain elevated for a brief period following *V. cholerae* infection (8). Because immunity to cholera persists longer than circulating *V. cholerae* antibodies can be detected, immunity may derive from a rapid anamnestic response of memory B cells generated from previous infections and/or the persistence of long-lived plasma cells at the mucosal surface. The former hypothesis has been supported by the observation that household contacts of cholera patients are better protected from infection if they have detectable levels of *V. cholerae* LPS-specific memory B cells at exposure, even if their levels of circulating vibriocidal antibodies are low (12).

Plasmablasts are activated antibody-secreting cells that are transiently found in the circulation after either infection or vaccination (13–16). We have previously demonstrated that cholera induces potent systemic plasmablast responses which can be readily detected 7 days after infection (17). A large proportion of these acutely induced plasmablasts express the gut homing receptor CCR9 (17). In addition, the magnitude of circulating *V. cholerae*-specific plasmablast responses on day 7 is strongly predictive of specific duodenal plasma cells for up to 6 months after cholera (18), suggesting that a proportion of these cells ultimately take up residence in the intestine as plasma cells, where they may mediate protection at the mucosal surface. Therefore, an in-depth characterization of circulating plasmablasts on day 7 following cholera provides a window into the properties of humoral memory both systemically and at the mucosal surface.

To that end, the aim of the present study was to investigate the response to *V. cholerae* infection by characterizing a panel of monoclonal antibodies (MAbs) generated by single-cell expression cloning of cholera-induced plasmablasts. Evaluation of the B cell response to cholera at a monoclonal level permits analyses that cannot be addressed by studying polyclonal responses. Specifically, this allows for an analysis of the origin of cross-reactivity between different *V. cholerae* serotypes, defining the repertoire breadth of responding B cells, and an assessment of the functional properties of the individual antibodies targeting *V. cholerae*. This approach also allows for an analysis of the cumulative effect of somatic hypermutation of *V. cholerae*-targeted antibodies. These are successively acquired, high-frequency point mutations that occur in the variable regions of the Ig heavy and light chains during B cell affinity maturation, which are typically associated with germinal center selection. The measurement of somatic hypermutation in antigen-specific cells may thus provide insights into how the B cell response to cholera occurs over time. In addition, human monoclonal antibodies against cholera may have translational implications for either diagnostic or therapeutic purposes. Finally, these studies may be instructive for future vaccine efforts against cholera, enterotoxigenic *Escherichia coli* (ETEC), and other mucosal infections.

## RESULTS

### Cholera induces a potent systemic plasmablast response.

**FIG 1:**
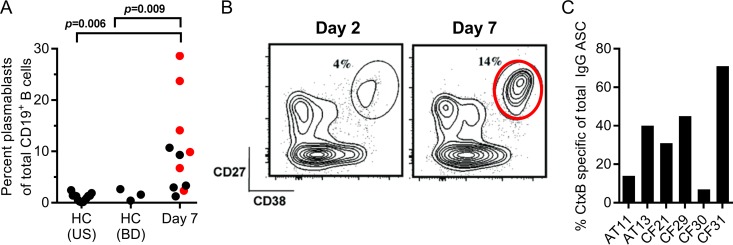
*V. cholerae* infection results in a potent and specific plasmablast response. (A) The proportion of circulating plasmablasts (CD3^−^ CD19^+^ CD20^−/low^ CD38^high^ CD27^high^) as a fraction of total CD19^+^ cells on day 7 following cholera. For comparison, the proportion of plasmablasts in healthy controls from both the United States and Dhaka, Bangladesh (BD), is shown. Single-cell-sorted plasmablasts from six donors (indicated in red) were used for the generation of monoclonal antibodies. (B) A representative flow cytometric analysis of plasmablast responses after cholera is shown where samples were collected at days 2 and 7 following hospitalization. Plasmablasts were isolated by cell sorting using the gate indicated in the day 7 flow plot. (C) The proportion of peripheral blood CtxB-specific IgG-secreting plasmablasts relative to the total number of IgG-secreting cells for the sorted samples as determined by ELISPOT assay. ASC, antibody-secreting cells.

We measured plasmablast responses in 11 adult men and women with severe cholera who presented at the International Centre for Diarrhoeal Disease Research, Bangladesh (icddr,b), hospital in Dhaka, Bangladesh, between 2011 and 2013. (Patient details are summarized in Table S1 in the supplemental material.) All patients were infected with the *V. cholerae* O1 Ogawa serotype, which accounted for 99.5% of cholera cases in Dhaka during the study period. Compared to healthy individuals without cholera from either an area where cholera is endemic (Dhaka, Bangladesh) or an area where it is nonendemic (United States), cholera induced a significant plasmablast expansion, representing up to 29% of the total B cells at day 7 following cholera (median, 9%; range, 1 to 29%; *P* = 0.009 compared to controls) (Fig. 1A). For six of the participants, we sorted plasmablasts into both bulk and single-cell populations (Fig. 1A and B). The bulk-sorted populations were used to confirm the presence of *V. cholerae* CtxB-specific plasmablasts by enzyme-linked immunosorbent spot (ELISPOT) assay, which ranged from 7 to 71% of the total IgG-secreting cell population (Fig. 1C), while the single-cell populations were used for the generation of human monoclonal antibodies as described below.

### Antibody specificity of the plasmablast response to *V. cholerae* infection at a single-cell level.

Using single-cell reverse transcription-PCR (RT-PCR), we isolated a total of 252 single-cell Ig amplicons from the sorted plasmablasts of six cholera patients (see Fig. S1 in the supplemental material). Analysis of these sequences showed a dominance of IgG-switched cells, followed by IgA and IgM, respectively. Overall, these sequences showed an average level of somatic hypermutation and clonality that was comparable to those of our previous studies of influenza and dengue infections (15, 16). From these amplicons, we generated a total of 138 recombinant human monoclonal antibodies (MAbs) (see Table S2 in the supplemental material), encompassing all of the IgG-secreting cells, as those cells made up a majority of the cholera-induced plasmablasts. In addition, we also included the entire set of clonal expansions, derived from antibody-secreting cells of all Ig isotypes. All antibodies, irrespective of the originating isotype, were expressed as IgG1 monoclonal antibodies.

**FIG 2:**
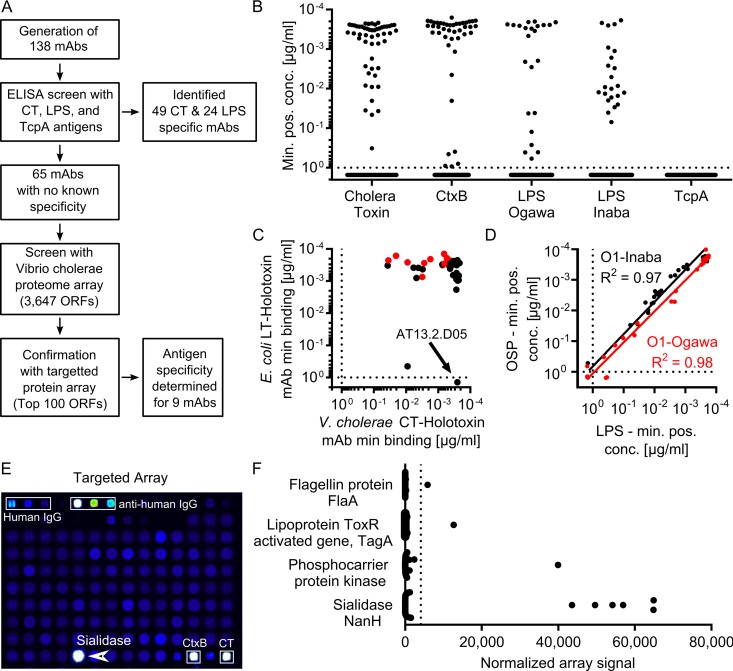
The majority of MAbs isolated from infected patients are specific for cholera toxin, LPS, or sialidase. (A) Flow chart describing experimental evaluation of MAb specificity. Antibodies were initially tested using an ELISA to assess binding to cholera toxin, LPS, and TcpA. Antibodies that did not bind to these antigens were subsequently examined using a novel *V. cholerae* proteome array. (B) Minimal binding concentrations to known *V. cholerae* antigens. Each antibody was measured in at least two independent experiments by ELISA to recombinant cholera holotoxin, recombinant CtxB, LPS derived from *V. cholerae* O1 serotypes Ogawa and Inaba, and TcpA. Values plotted represent average minimal effective concentrations, determined as the minimum MAb concentration required for 3 times the background signal of sample dilution buffer. (C) The minimal effective concentration for binding to CT holotoxin (*x* axis) and LT holotoxin (*y* axis). (D) An XY scatter plot shows the average minimum positive concentration for binding to OSP (*y* axis) and LPS (*x* axis) for serotypes O1-Ogawa (red) and O1-Inaba (black) as determined by two independent ELISA experiments. Solid lines represent the best-fit linear regression line of log-transformed values for each serotype. Dotted lines represent the maximal concentration tested for each MAb. (E) Representative data output showing the targeted array. (F) Summary of all the MAbs with unknown specificity as evaluated by the targeted miniarray. The dotted line represents a threshold signal set at 10 times the array signal intensity range of an isotype control antibody (EM4C04, influenza virus hemagglutinin [HA]-specific IgG1). Only antigens with at least one binding MAb are shown.

To determine the antigen specificity of the monoclonal antibodies generated, we used a combination of screening against known immunodominant antigens and a novel proteomic-based screening approach to define the antigenic target of MAbs with unknown specificity (approach outlined in Fig. 2A). Initially we measured the affinity of each MAb in the panel against the well-characterized *V. cholerae* antigens CT, LPS, and TcpA (Fig. 2B). Of the 138 antibodies, 24 bound to *V. cholerae* O1 LPS (either the Inaba or Ogawa serotype or both), and 49 bound to CT. Although TcpA has been identified as a target of the *V. cholerae* infection-induced immune response, and leads to the generation of long-lived memory B cells (11), none of the antibodies we generated bound to TcpA (Fig. 2B).

The majority of CT-specific antibodies bound to the CtxB subunit with high affinity. The minimum effective concentration of MAb detectable by enzyme-linked immunosorbent assay (ELISA) was less than 1 ng/ml for 35 of the 37 (95%) CtxB-specific antibodies (see Fig. S2 in the supplemental material). In addition, we identified 12 antibodies that bound the CT holotoxin but did not target CtxB (Fig. S2; marked in red). This suggests that a subset of antitoxin responses target either the CtxA subunit or a conformational epitope only present in the active holotoxin. Most of the identified CT-specific MAbs were cross-reactive with the closely related enterotoxigenic *E. coli* heat-labile toxin (LT) (Fig. 2C). In fact, only two of the CT-specific MAbs did not cross-react with LT, including one of the highest-affinity CT-binding antibodies (MAb AT13.2.D05). Interestingly, many antibodies bound with higher affinity (>5-fold) to LT than CT, including 5 of the 38 anti-CtxB antibodies and 6 of the 12 anti-CtxA/holotoxin antibodies (Fig. 2C), suggesting that there is extensive cross-talk between the immune responses to the highly conserved CT and LT toxins.

Of the 138 antibodies generated, 24 bound to *V. cholerae* O1 LPS. LPS consists of three domains: lipid A, a core oligosaccharide, and the variable O-specific polysaccharide (OSP). While all three of these domains are targeted *in vivo* in mice immunized with LPS (19), it is less clear what the immune response is focused on in a human infection. To define the binding epitopes recognized by human antibodies to *V. cholerae* LPS, we compared the affinities of our LPS-specific MAbs with that of purified OSP that was conjugated to bovine serum albumin (BSA). The binding affinities for LPS correlated very well with OSP binding for both serotypes (*R*^2^ = 0.97 for Inaba, and *R*^2^ = 0.98 for Ogawa), and all antibodies that bound to LPS at minimal effective concentrations of less than 0.1 µg/ml also bound to OSP (Fig. 2D). This demonstrates that the immune response against *V. cholerae* LPS almost exclusively targets the OSP, rather than the lipid A or core oligosaccharide moieties.

Finally, in an effort to define the specificity of the remaining 65 monoclonal antibodies that did not bind to CT or LPS, we generated and probed a *V. cholerae* proteome microarray with these MAbs. The full array included 3,647 open reading frames (ORFs), and the antibodies were pooled and tested in groups of 9 MAbs per protein array. This analysis along with ongoing serum studies (data not shown) identified a subset of 100 potential antigens, which were subsequently printed on a targeted secondary array on which each MAb was then assessed. This analysis identified nine human MAbs that bound to novel *V. cholerae* antigens (Fig. 2F). Six of these MAbs bound to the *V. cholerae* sialidase (NanH) (Fig. 2E). We also identified the target of three additional antibodies, including a protein kinase, flagellin protein A (FlaA), and the ToxR-regulated mucinase TagA (Fig. 2F). The specific binding of each MAb to these novel target antigens was confirmed by a dose-response analysis, showing that variable concentrations of these antibodies demonstrated the first-order binding kinetics characteristic of a specific antigen-antibody interaction (see Fig. S3 in the supplemental material).

### High levels of somatic hypermutation in O1 LPS-specific plasmablasts correlate with serotype specificity.

**FIG 3:**
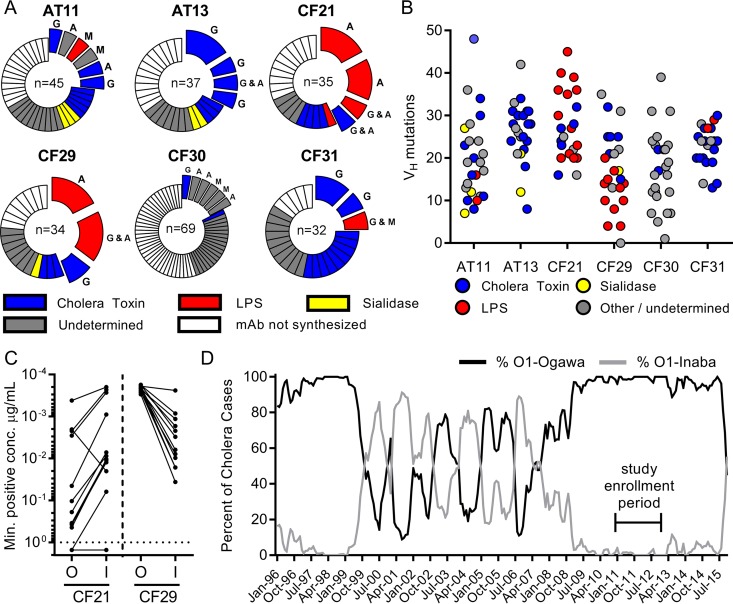
Identification of LPS-specific MAbs in clonally expanded cells that have marked heterogeneity in somatic hypermutation and affinity toward a historical *V. cholerae* serotype. (A) The repertoire breadth of plasmablast-derived heavy chain sequences is indicated. For each patient, the number at the center of each pie chart indicates the number of heavy-chain sequences evaluated. Clonal expansions (defined as two or more sequences sharing the same VDJ rearrangement and junctional diversity) are shown as expanded sections of the pie chart. Colors denote antibody specificity as described in the panel legend. The Ig isotypes identified within each clonal expansion are shown adjacent to each section (G, IgG; A, IgA; M, IgM). (B) A comparison of Ig heavy-chain somatic hypermutation, relative to the closest germline sequence, with antibody specificity. (C) Antibody affinity to LPS as measured by ELISA is shown as the minimum MAb concentration required for 3 times the background signal of sample dilution buffer. (D) Epidemiological prevalence of the O1 serotypes Ogawa and Inaba in the patient cohorts at icddr,b in Dhaka. Our study was conducted during an extended period with an extremely low prevalence of the Inaba serotype.

Based on V_H_ gene sequence analysis, an average 31% of the cells we analyzed originated from clonally expanded B cells, with the overall observed clonality ranging from 17 to 44% among the study participants (Fig. 3A). As expected, the clonally expanded plasmablast populations were highly enriched for *V. cholerae* antigen-specific cells, and over 75% of the antibodies derived from clonally expanded plasmablasts bound to either CT or the *V. cholerae* LPS antigen (Fig. 3A). Analysis of the V_H_ sequences also revealed high levels of somatic hypermutation, with an average number of mutations per V_H_ gene ranging from 17 to 24 (standard deviation [SD] of 3) V_H_ mutations across study participants (Fig. 3B). The CT-specific cells were uniformly heavily mutated in all participants, containing an average of 23 mutations per V_H_ gene across all donors. One example of a key point mutation within clonal expansions of CT-specific MAbs was identified that was able to raise the affinity to CT more than 20-fold based on a single mutation (data not shown). Interestingly, while the CT MAbs displayed a relatively homogeneous number of mutations, the LPS-specific MAbs displayed a mutational load that varied widely between study participants. Comparison of the LPS-specific responses of subjects CF21 and CF29 is especially informative. In both subjects, we observed large clonal expansions of LPS-specific plasmablasts, made up predominantly of IgA-expressing cells (Fig. 3A). While both subjects were infected with the Ogawa serotype, the LPS-specific antibodies derived from patient CF21 more effectively bound to and killed the previously circulating Inaba serotype (Fig. 3C). The CF21-derived antibodies were also characterized by extremely high levels of somatic hypermutation, with an average of 30 mutations per V_H_ gene (including some antibodies with >40 mutations). In contrast, the antibodies specific for LPS in donor CF29 carried an average of 13 mutations per V_H_ gene and preferentially bound to and killed the infecting Ogawa serotype (Fig. 3C). These findings suggest that the current Ogawa infection triggered a response characterized by the recall of cross-reactive memory B cells originally induced by previous infection with the Inaba serotype. This is remarkable given that the Inaba serotype had been rarely detected in Dhaka during the preceding 4 years (Fig. 3D). In addition, within the most highly mutated clonal expansions in patient CF21, all antibodies predominantly bound the Inaba serotype, while a recently emerged variant showed increased binding to the Ogawa serotype (CF21.2.F1) (see Fig. S4 in the supplemental material).

### Functional characterization of the human antibody responses to cholera at the single-antibody level.

**FIG 4:**
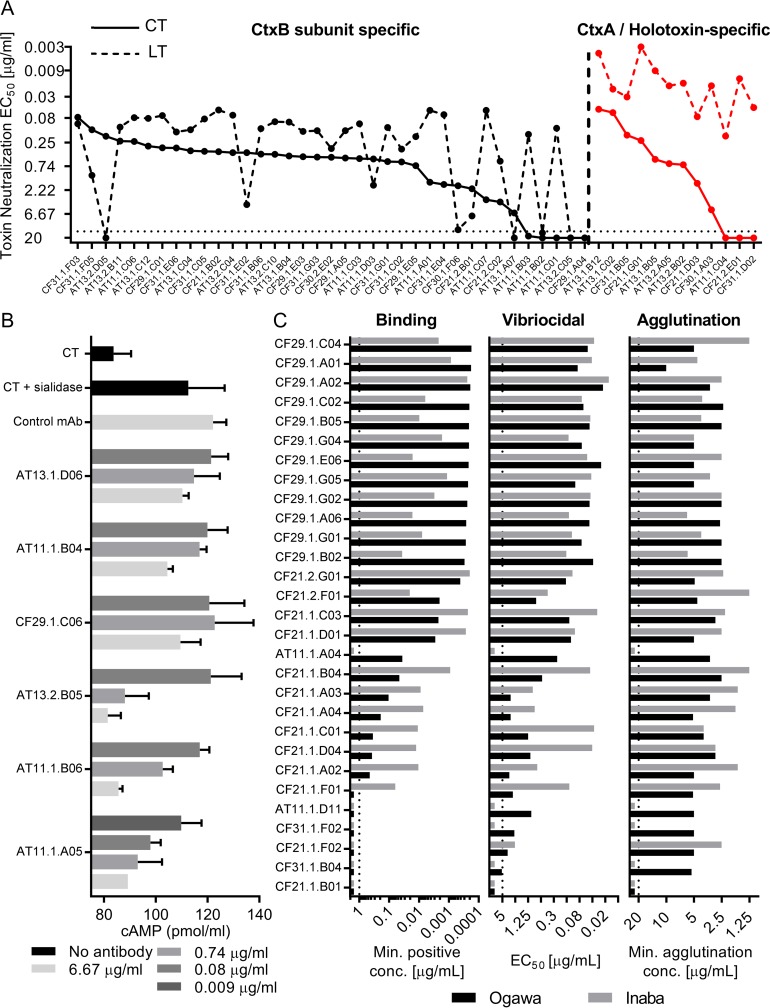
Functional evaluation of antigen-specific MAbs. (A) Comparison between CT and LT neutralization 50% effective concentration (EC_50_) values for each CT-specific antibody. Values (*y* axis) are the toxin neutralization EC_50_ values derived from the mean of two technical replicates. Values for CT are connected by a solid line, while those for LT are connected by a dashed line. In decreasing order of neutralization potency, CtxB subunit-specific antibodies are shown to the left of the vertical dashed line, while CtxA/holotoxin-specific antibodies are shown to the right (in red). (B) Sialidase treatment enhanced the effect of CT-induced cAMP production of cultured Caco-2 cells compared to that of a CT-only control. Sialidase-specific MAbs neutralized this enhancing effect in a dose-dependent manner. Data and standard error of the mean (SEM) values are derived from three technical replicates. An LPS-specific MAb was used as a negative control for this experiment (CF29.1.B02). (C) Comparison of binding affinity, vibriocidal activity, and agglutination potency for all LPS-specific antibodies, arranged according to Ogawa binding potency. For binding assays, bars represent the average minimum MAb concentration required for 3 times the background signal of sample dilution buffer (*x* axis). The dotted line marks the highest concentration of antibody evaluated (1 µg/ml). The five MAbs that have minimal effective concentrations of >1 µg/ml were derived from clonal expansions containing other LPS-specific antibodies. Binding, agglutination, and vibriocidal experiments were performed in three independent experiments. Values represent experimental averages.

While it is established that cholera immune sera can neutralize cholera toxin, it is not clear what fraction of antibodies are able to do this or how potent they are individually. In addition, while cross-reactivity to the homologous ETEC heat-labile toxin (LT) is evident at a serological level, it remains unclear to what degree the human antibody response against CT cross-reacts with LT at a monoclonal level. To assess this, we quantified the toxin neutralization capacity of these MAbs against both CT and LT. Both CtxB-specific and CtxA/holotoxin-specific antibodies were able to neutralize CT. The vast majority of the MAbs were in fact able to neutralize the toxin, including the CtxA/holotoxin-specific ones. In fact, the two antibodies that showed the highest CT-neutralizing potency *in vitro* were of this category of CtxA/holotoxin-specific antibodies (Fig. 4A). This suggests that antibodies targeting either CtxA or holotoxin-specific epitopes may play a functional role in the human immune response to cholera. In addition, most of the MAbs could also effectively neutralize LT, as 37 of the 49 anti-CT antibodies were potent neutralizers of LT, and all 12 antibodies targeting the CtxA-subunit/CT-holotoxin effectively neutralized LT with very high potency (Fig. 4A). In fact, we could only identify a single potent and truly CT-specific MAb, while there were a number of LT-specific potent neutralizers, especially from the CtxA/holotoxin-specific group.

As described above, we identified several *V. cholearae* sialidase-specific antibodies using proteomics approaches. *V. cholerae* sialidase modifies host glycolipids, potentially increasing the availability of the CT receptor GM1 ganglioside on the cell surface (20). To assess whether these antibodies could also have a functional role in protecting against cholera, we tested them in an assay measuring cholera toxin-induced cyclic AMP (cAMP) production in the human colorectal Caco-2 cell line. We found that *V. cholerae* sialidase could potentiate the effect of CT alone, by an average of 35% (Fig. 4B). All six of the sialidase-reactive antibodies could reduce this potentiating effect, and three of the six were able to completely neutralize the toxin-potentiating effect of sialidase, suggesting that these antibodies could also have a physiologic role in the human immune response to *V. cholerae* infection (Fig. 4B).

Because the vibriocidal antibody response is strongly associated with protective immunity, we evaluated the vibriocidal capacity of each MAb, assessing the ability of each antibody to induce complement-mediated killing of *V. cholerae* O1. We also measured the agglutinating titer of each MAb, as agglutination might play a physiologic role in blocking or clearing infection at the mucosal surface (21). For these assays, we tested both the LPS-specific MAbs as well as MAbs with unknown antigenic specificity, and interestingly, only the MAbs that bound to LPS demonstrated any vibriocidal or agglutinating capacity (Fig. 4C), suggesting that these physiologically important responses are primarily derived from O-antigen-specific responses. Figure 4C shows five MAbs that do not appear to bind to LPS by the ELISA shown. Of these, all five antibodies were part of clonal expansions containing other LPS/OSP-specific antibodies, and three were positive for purified OSP.

While the affinity of the antibodies for LPS largely correlated with vibriocidal capacity in a serotype-dependent fashion, the bacterial agglutination ability of the individual antibodies did not correlate with affinity or vibriocidal measures (Fig. 4C; see Fig. S5 in the supplemental material). In particular, several potent agglutinating antibodies in the panel bound poorly or not at all to LPS and were only weakly vibriocidal. This might not be unexpected, given that for agglutination the avidity of a large number of antibodies, rather than the affinity of individual antibodies, may be a more important measure. It is also interesting that while the majority of the antibodies are cross-reactive, they nonetheless demonstrate preferential binding to one serotype over the other. Of note, a small number of antibodies, however, showed complete specificity to the Ogawa serotype and did not react with the Inaba strain at all for any of the functional measures performed (i.e., AT11.1.A04). Taken together, these data suggest that these physiologically important antibody responses are almost exclusively derived from O-antigen-specific responses, which may have implications for our understanding of protective immune responses against cholera and aid in guiding future vaccine design.

## DISCUSSION

Antibodies are thought to be the main mediator of the immune protection induced after natural *V. cholerae* infection. Thus, it is imperative to understand the properties of the infection-induced antibodies in more detail, establish novel correlates of long-lived immune protection, define the functional properties and cross-reactivity between different *V. cholerae* serotypes, and understand how long-lived immune protection can be induced by natural infection. In the present study, we analyzed the immunoglobulin repertoire, specificity, and functional characteristics of a panel of monoclonal antibodies generated from cholera-induced, systemic plasmablasts. While this approach has been successfully used to study a number of systemic viral infections in humans, such as, for example, influenza and dengue (13, 14, 16), these approaches have not been widely applied to human bacterial infections. To our knowledge, only one other study has analyzed the antibody repertoire of the plasmablast response to a human bacterial infection, demonstrating that invasive *Staphylococcus aureus* infection was dominated by the expansion of the V_H_3 idiotype driven by the superantigen protein A (22). In our study, we found that mucosal infection with cholera induces a potent systemic plasmablast response, characterized by a high degree of clonality, somatic hypermutation, and a restricted repertoire against a handful of dominant antigens. Surprisingly, the degree of clonal expansion and somatic hypermutation we observed in this study following cholera was comparable to, or even more extensive than, what we have previously observed for both influenza and dengue acute viral infections in a recall response setting (13, 14, 16). Interestingly, the data presented herein raise interesting questions about the degree of cross-reactivity not only between different cholera strains, but also between cholera and other enteric infections. These findings may have important implications for our understanding of mucosal immune responses and how these responses may differ in regions of endemicity and nonendemicity. This in turn may provide important insight for future rational vaccine design.

### Limited breadth of antigen recognition by the immune response against cholera.

*V. cholerae* produces a large number of potential antigens, including bacterial outer membrane structures, secreted proteins, and exopolysaccharides, which interact with the host environment and are implicated in the pathogenesis of cholera. Yet, despite the number and complexity of potential antigens produced by *V. cholerae*, we found that the human immune response to cholera is targeted against a select number of immunodominant components. The observation that 52 of 68 (Fig. 3A), or approximately 75%, of the antibodies derived from clonally expanded plasmablasts targeted either CT or LPS provides compelling evidence for the dominance of these antigens and demonstrates the selective targeting of the human immune in an otherwise antigenically complex mucosal milieu.

The limited number of *V. cholerae* antigens makes the identification of sialidase as a novel target of the human immune response notable. Like CT, *V. cholerae* sialidase is secreted by a type II secretion system and interacts directly with the host enterocyte. *V. cholerae* sialidase converts higher-order gangliosides into GM1, the receptor for CT, by removing sialic acid residues (20, 23). The sialidase-encoding *nanH* gene is located on *Vibrio* pathogenicity island 2 (VPI2), a genomic island uniformly present in pathogenic *V. cholerae* but typically absent in nonpathogenic *Vibrio* (24). While these features suggest a key role of the *V. cholerae* sialidase in the pathogenesis of cholera, sialidase was not shown to potentiate the effects of CT in a rabbit ileal loop model of cholera (23) and had a modest effect on cholera pathogenesis in a suckling mouse model (20). These studies have led to speculation that sialidase may play a limited role in the pathogenesis of cholera. In our study, sialidase augmented CT responses *in vitro*, a potentiating effect that, importantly, could be fully neutralized by sialidase-specific human monoclonal antibodies. Thus, our findings support a role for *V. cholerae* sialidase in host-pathogen interactions in human cholera.

Although we identified the target of the majority of the synthesized MAbs (56%), particularly of the clonally expanded fraction (75%), the antigenic target for some of the MAbs remain undetermined. There are several limitations of our study that may account for this. First, our proteomics-based antigen screening most likely did not reflect the full spectrum of *V. cholerae* antigens targeted by the cholera-induced plasmablast response. For example, some of the *in vitro-*translated proteins produced on the array may have been poorly expressed, folded incorrectly, or were incorrectly glycosylated, thus preventing identification. Second, because *V. cholerae* infection occurs at a nonsterile site, it is possible that some of the immune response following cholera may target other microbes that are present at the mucosal surface during cholera, such as other bacterial flora or viruses (including bacteriophages). Finally, because some circulating plasmablasts are observed at baseline in otherwise healthy individuals, it is likely that some of the plasmablasts in our sample were not related to the cholera-induced immune response. Despite these limitations, our approach offers several advantages compared to other alternatives available to characterize the B cell compartment after bacterial infection. Specifically, by isolating and characterizing *in vivo* activated cells, it allows for a nonbiased analysis of the pathogen-driven immune response. In contrast, alternative approaches for generating human monoclonal antibodies based either on Epstein-Barr virus (EBV)-mediated immortalization or flow-based cell sorting of single, antigen-specific memory B cells rely on a binding or functional screen or the utilization of a labeled antigen probe. While this is both attractive and feasible for many viral antigens, such approaches may be less optimal for a complex bacterial pathogen.

### Cholera toxin responses are likely influenced by recall responses from prior exposure to ETEC LT.

Surprisingly, we found that 85% of the CT-specific monoclonal antibodies were able to neutralize the toxin *in vitro*. Almost all of these antibodies were also able to neutralize LT, which shares 80% homology with CT at the amino acid level, with the exception of the region around the A1 and A2 cleavage, where the homology decreases to 33% (25, 26). In fact, taken together, 46/49 (94%) of the CT reactive antibodies could neutralize either CT, LT, or both, a diverse set which included a large number of antibodies targeting CtxB as well as other epitopes present on either CtxA or the CT holotoxin.

Prior to this study, we would have predicted that only subset of the CtxB-specific antibody would have effectively mediated toxin neutralization. This is because antibody-mediated toxin neutralization is generally achieved by inhibiting toxin-receptor binding. In addition, previous studies in animal models have suggested that CtxB is a stronger immunogen than CtxA (27, 28). For example, a study of 61 monoclonal antibodies derived from CT-immunized mice demonstrated that while CtxB-specific antibody responses were dominant and protective, the CtxA-specific antibodies demonstrated no appreciable toxin-neutralizing capacity (27). Furthermore, only 20% of the mouse antibodies were equally reactive with the ETEC LT toxin, and most did not bind to LT (27). These studies supported the prevailing view that the CtxA subunit does not contribute protective antigenic determinants; a conclusion that has significantly impacted the design of oral cholera vaccines. For example, the currently licensed cholera vaccines that contain or express the CtxB subunit at high levels include Dukoral (WC-rCTB), a killed vaccine, and Vaxchora (CVD 103-HgR), a live attenuated vaccine approved by the U.S. FDA in June 2016. In contrast, no current cholera vaccine includes a CtxA or holotoxin-based toxoid derivative.

While our results confirm CtxB is a dominant antigen in humans, we found that responses to CtxA or unique conformational structures in the holotoxin represent a substantial fraction of the antitoxin response and that two of the most potent toxin-neutralizing antibodies fell in this category. Thus, these responses also likely contribute to the functional antitoxin response in humans. From a translational standpoint, it is especially notable that these holotoxin-specific antibodies were also remarkably effective at neutralizing the LT toxin, suggesting that antibodies targeting the CtxA subunit may be better able mediate cross-protection against both cholera and ETEC. This is consistent with recent findings demonstrating that human ETEC infection results in a substantial fraction of serum antibodies that target the EltA subunit and neutralize LT (29). Furthermore, a CtxA/EltA-derived toxoid-based vaccine may also avoid the unwanted immunomodulatory effect of CtxB, which has been postulated to account for the observation that killed whole-cell vaccines that do not contain recombinant CtxB may provide longer-lived protection than killed whole-cell vaccines that do include CtxB (30, 31). The activation of cross-reactive memory B cells initially induced by a prior exposure to the LT antigen is the most likely explanation for why our results differ from those of previous studies in animals. Prior exposure to ETEC infection is likely to be nearly ubiquitous in most areas where cholera is endemic. The fact that most of the CT-specific MAbs bound to and neutralized LT provides strong evidence that these responses were shaped by high levels of previous exposure to this similar antigen.

### Responses to the *V. cholerae* LPS demonstrate expansion of cross-reactive memory B cells against the O1-specific polysaccharide antigen.

In this study, we also describe the first characterization of the human response to *V. cholerae* O1 LPS at the monoclonal antibody level. Our results demonstrate that the majority of the cholera-induced response to LPS is directed against the O-specific polysaccharide (OSP) moiety of LPS (Fig. 2C). This is notable because studies of LPS responses to inactivated *Salmonella enterica* in mouse models suggest that both O-antigen and lipid A moieties are dominant antigenic determinants of LPS (19, 32). A study of the host-restricted mucosal pathogen *Helicobacter pylori* demonstrated that human IgA and IgM responses target the lipid A moiety of LPS, while IgG responses primarily target the variable O-specific polysaccharide (33). In contrast, our results suggest that in *V. cholerae* infection, the antibody response to LPS is almost exclusively directed at the O-specific polysaccharide. Furthermore, our data show that both vibriocidal antibody responses and bacterial agglutination responses are functional aspects of the cholera antibody response that are derived from OSP-binding MAbs.

We also found that these *V. cholerae* OSP-specific antibodies displayed variable serotype specificity and varied significantly with respect to functional qualities in terms of bactericidal activity and agglutination. The range of variation in *V. cholerae* O1 serotype specificity in individual LPS-binding antibodies was remarkable given that the structural difference between the Ogawa and the Inaba antigens is only a single methyl group at the terminal sugar. This variation encompassed a spectrum ranging antibodies binding Inaba only to those binding Ogawa only, although most antibodies displayed some degree of cross-reactivity. This finding provides a mechanistic basis for the partial heterotypic cross-protection that has been inferred from epidemiological modeling of fluctuations in serotype prevalence (34).

Finally, the observation that the cholera-induced plasmablast response to the *V. cholerae* O antigen in some patients was characterized by very high levels of somatic hypermutation and likely the result of a recall response of memory B cells from a prior antigen exposure was an unexpected finding. These features are suggestive of both long-term memory and germinal center responses with extensive affinity maturation to the *V. cholerae* O1 polysaccharide antigen. It is also notable that LPS-specific antibodies were largely absent in our comprehensive panel of antibodies derived from IgG-secreting plasmablasts, while CT-specific antibodies were ubiquitous. Murine data have suggested that IgA class-switched responses to polysaccharides in the intestinal mucosa can occur directly in the lamina propria through T-cell-independent mechanisms, including *TNFRSF13B*-induced activation of activation-induced cytidine deaminase (AID) (35, 36). However, it has not been previously reported that T-cell-independent IgA class switch pathways result in the high levels of somatic hypermutation to the extent we have demonstrated in response to the *V. cholerae* O1 antigen, making this explanation improbable.

In fact, the extent of hypermutation and the recall of prior antigenic exposure that we have observed are distinct from the type of IgA memory induced by transient mucosal colonization by bacteria in mice, which does not result in a prime-boost effect following reexposure to bacterial antigens (37). Thus, our findings raise important questions about whether there are specific pathways that are critical for the development of highly refined, long-lasting IgA memory B cells that are specific to the polysaccharide antigens of intestinal pathogens and whether these responses are germinal center independent or not. Further delineation of these mechanisms may have implications for the development of mucosal vaccines to polysaccharide antigens.

### Concluding remarks.

Our study represents the first examination of the human B cell response to cholera at a single-cell/single-antibody level. While similar studies of the human B cell repertoire have been applied to viral infections (e.g., influenza, dengue fever, and HIV), this is only the second time such an approach has been described for a bacterial infection. Because we conducted this study in adults living in an area where cholera is endemic, our findings provide insights into the natural history of infection with *V. cholerae* O1. Specifically, we found strong evidence that cross-reactive B cell memory between the CT and LT toxins and between the Inaba and Ogawa serotypes influences the response to cholera in this setting. Similarly, it is possible that variation in previous exposures to *V. cholerae* may explain the heterogeneity in antibody responses we observed between participants. Studies in young children, in volunteers from areas of nonendemicity, and in cholera vaccine recipients may increase our understanding of the initial antibody response to cholera, the duration of B cell memory, and how these responses are honed over time in different populations.

Our findings also elucidate several novel aspects of the interaction between the human-restricted pathogen *V. cholerae* and its natural host, including the recognition of new antigenic targets of the human immune response to this globally important pathogen. We also demonstrate that key functional aspects of the cholera response, including vibriocidal antibody responses and bacterial agglutination responses, are completely derived from OSP-binding MAbs. From a methodological standpoint, our ability to both identify the antigenic target and assess antibody function, for the majority of the acutely activated B cells isolated following cholera, suggest that this type of approach can be applied to understand the human immune response to other bacterial pathogens, vaccines, and possibly more complex interactions between the human host and the intestinal microbiome.

## MATERIALS AND METHODS

A detailed materials and methods section is provided in Text S1 in the supplemental material.

### Patient enrollment.

Participants with confirmed *V. cholerae* O1 infection were enrolled between 2011 and 2013 at the International Centre for Diarrhoeal Disease Research, Bangladesh (icddr,b) hospital in Dhaka, Bangladesh. Participants provided written informed consent, and the study was approved by the Institutional Review Board (IRB) in both Dhaka and at Massachusetts General Hospital.

### Flow cytometry and single-cell sorting.

Blood was collected by venipuncture during acute infection (day 2, or the second day of hospitalization) and during convalescence at day 7. Peripheral blood mononuclear cell (PBMC) preparation and both analytical flow and single-cell sorting were done as previously described (13, 17, 38).

### Generation of recombinant monoclonal antibodies.

MAbs were generated from single-cell-sorted plasmablasts by single-cell expression cloning, essentially as previously described (13, 38) with minor modifications (described in Text S1). In brief, heavy- and light-chain Ig rearrangements were identified from single-cell cDNA, cloned into human expression vectors, produced by transient transfection of Expi293F cells, and purified using protein A beads.

### Sequence analyses.

Sequence analysis was performed as previously described (13, 16).

### Antigens.

LPS was prepared as previously described (39). OSP was purified and conjugated to bovine serum albumin (BSA) as previously described (39–41). CT, LT, and EltB were provided by Clemens and prepared as previously described (42). Recombinant TcpA was prepared as previously described (43). Purified recombinant CtxB (Reagent Proteins) and purified *V. cholerae* sialidase (Roche) were purchased from commercial vendors.

### ELISAs.

ELISAs for CtxB, LT, EltB, and TcpA, as well as bovine serum albumin (BSA)-conjugated O-antigen-specific polysaccharide (OSP) were performed essentially as previously described (11).

### *V. cholerae* protein microarray analysis.

*V. cholerae* proteome microarrays were produced using genomic DNA (gDNA) from the reference strain N16961 as template. A total of 3,647 ORFs larger than 150 bp were expressed by *in vitro* translation and spotted onto nitrocellulose-coated glass slides as previously described (44). These arrays were initially probed with pools of 9 MAbs of unknown specificity. A targeted microarray was then fabricated containing the top 100 cholera ORFs giving detectable signal in this experiment or when screened by serum. The targeted array was used to screen individual MAbs. For identified target antigens, different concentrations of individual MAbs ranging between 0.001 and 100 μg/ml were titrated on the microarray to generate binding curves.

### Toxin and sialidase neutralization assays.

CT and LT neutralization assays were essentially performed as previously described (29). To measure the CT-potentiating effect of sialidase and to test the impact of the MAbs on this, MAbs were serially diluted in phosphate-buffered saline (PBS) and mixed with 2 mU/ml *V. cholerae* sialidase. After incubation, the mixture was added to Caco-2 cells for 1 h, and the cells were washed, stimulated with CT, and processed as described above.

### Vibriocidal and agglutination assays.

Vibriocidal and agglutination assays were performed with the *V. cholerae* strains O1-Ogawa (X25049) and O1-Inaba (T19479). Vibriocidal assays were conducted as previously described (11). A quantitative bacterial agglutination assay using nonfixed bacteria was developed (described in detail in the supplemental material) and used to measure agglutinating titers.

## SUPPLEMENTAL MATERIAL

Text S1  Supplemental materials and methods. Download Text S1, PDF file, 0.1 MB

Figure S1  *V. cholerae* infection results in a potent and specific plasmablast response that has undergone isotype switching, somatic hypermutation, and clonal expansion. (A) The immunoglobulin isotype frequency of single cells as determined by sequencing. Numbers in parentheses represent the total number of single cells analyzed. (B) The mean number of somatic mutations in the V_H_ gene for each study participant is shown; bars represent median values. Published historical results from previous studies of influenza and dengue fever are provided for comparison (14, 16). (C) The percentages of Ig sequences from each patient that were derived from clonally related cells, as indicated by shared V_H_ and J_H_ segments, as well as CDR3 junctional diversity are shown, with comparisons to previously published data on systemic viral infections; bars represent median values (14, 16). Download Figure S1, PDF file, 0.1 MB

Figure S2  Cholera toxin-specific antibodies. The antibody affinity to cholera holotoxin and CtxB is shown. Antibodies denoted by red bars bound to CT holotoxin but not the CtxB subunit at a concentration of 1 µg/ml (dotted line). Each antibody was measured in at least two independent experiments. Download Figure S2, PDF file, 0.1 MB

Figure S3  *V. cholerae*-specific MAbs show a dose-dependent binding to four antigens using a protein microarray. Binding curves of sialidase, FlaA-, TagA-, and phospho-carrier protein kinase-specific MAbs were generated using the targeted array. Different concentrations of antibody ranging from 100 to 0.001 µg/ml were titrated on microarray chips. For all antibodies, the signal intensity was antibody concentration dependent. Download Figure S3, PDF file, 0.2 MB

Figure S4  Expansion of highly mutated LPS-specific antibodies that preferentially recognize a currently noncirculating *V. cholerae* strain. (A) For three study participants, the plasmablasts derived from clonally expanded populations are grouped and shown as expanded sections in the pie charts. Clonal expansions (CE) containing LPS-specific antibodies are shown in red and numbered to correspond to specific comparisons between clonal expansions described below. (B) Antibody affinity by ELISA to LPS is shown as the minimum MAb concentration required for 3 times the background signal of sample dilution buffer. (C) EC_50_s corresponding to the vibriocidal activity of each MAb are shown. In panels B and C, MAbs belonging to a unique clonal expansion are grouped. The asterisk denotes antibody CF21.2.F1. **(**D**)** V_H_ mutations observed for CT- and LPS-specific antibodies. Clonal expansions for LPS-specific antibodies are denoted as follows: group 1, circles; group 2, squares; group 3, triangles. (E) Phylogram generated by maximum likelihood analysis of heavy-chain variable domain sequences derived from the highly mutated LPS-specific clonal expansion no. 1 observed in patient CF21. Download Figure S4, PDF file, 0.2 MB

Figure S5  Comparative analysis of vibriocidal and agglutination functional characteristics (A and B). The strains and LPS used in these assays were derived from *V. cholerae* O1-Ogawa (left) and O1-Inaba (right). Lines represent linear regression analysis of log_10_-transformed values. Outliers that were below the limit of detection in both assays were excluded from regression analyses. (A) Correlation between vibriocidal EC_50_ values (*y* axis) and the minimum positive binding concentration in an ELISA (*x* axis). (B) Correlation between the minimum agglutination antibody concentration (*y* axis) and the minimum positive binding concentration in an ELISA (*x* axis). (C) Representative analysis of antibody-mediated vibriocidal activity is shown to *V. cholerae* O1-Ogawa for MAb AT11.1.A04. Bars show SEM of the assay measured in triplicate. Higher values on the *y* axis correspond to increased culture turbidity as measured by UV absorbance at 600 nm. EC_50_s were determined as the concentration of MAb that effected a 50% reduction in bacterial growth (dotted line). (D) Representative analysis of antibody-mediated bacterial agglutination with 2-fold titration of the MAb shown. White arrows depict the well displaying the minimal agglutination concentration. Download Figure S5, PDF file, 0.2 MB

Table S1  Cholera patient cohort data. Table S1, PDF file, 0.2 MB

Table S2  Summary of monoclonal antibody panel. Table S2, PDF file, 0.2 MB

Table S3  Summary of MAb specificity. Table S3, PDF file, 0.2 MB

Table S4  Summary of monoclonal antibody characteristics. Table S4, PDF file, 0.2 MB
