# Supplementary material for: Single-Cell Analysis of the Plasmablast Response to Vibrio cholerae Demonstrates Expansion of Cross-Reactive Memory B Cells
Source: mBio. 2016 Dec 20;7(6):e02021-16. doi: 10.1128/mBio.02021-16 (PMC5181778; doi:10.1128/mBio.02021-16)
Supplement: Text S1 — Supplemental materials and methods. Download [file mbo006163110s1.pdf]

SUPPLEMENTAL MATERIAL

1. Supplemental Materials and Methods

Patient enrollment. All participants were enrolled between 2011-2013 at the International Centre for Diarrhoeal Disease Research, Bangladesh (icddr,b) hospital in Dhaka, Bangladesh. Participants with severe diarrhea due to *V. cholerae* O1 infection, confirmed by stool culture, were included in this study (1). All participants were negative for ETEC by stool culture (2). All participants provided written informed consent, and the study was approved by the Research Review and Ethical Review Committees of the icddr,b, Dhaka, Bangladesh, and the Institutional Review Board (IRB) of the Massachusetts General Hospital.

Sample collection and processing. 10 ml of whole blood was collected by venipuncture during acute infection (day 2, the second day of hospitalization) and during convalesce at day 7. PBMCs were isolated by differential centrifugation on Ficoll-Isopaque (Pharmacia, Piscataway, NJ), then washed, counted, and suspended at a concentration of 1 x 10^7 cells/ml in RPMI complete medium (Gibco, Carlsbad, CA) supplemented with 10% heat-inactivated fetal bovine serum (FBS) (HyClone, Logan, UT).

Flow cytometry and single cell sorting. PBMC staining for analytical flow cytometry analyses was performed as previously described (3). PBMCs were stained with CD3 Pacific blue (BD Pharmingen, USA), CD19 FITC (BD Pharmingen, USA), CD20 PE Cy7 (BD Biosciences, USA), CD27 APC (eBioscince, USA), CD38 PE (BD Pharmingen, USA)CD3 Amcyan (BD Biosciences, USA), CD 19PE-Texas Red (Invitrogen,USA), CD20 FITC (Invitrogen,USA), CD27APC-Cy7 (Biolegend, USA), and CD38 PE-CY7 (BD Biosciences, USA. Plasmablasts were defined as CD3-/$CD19^{+}$/$CD20^{-/low}$/$CD27^{high}$/$CD38^{high}$ cells. Single-cell and bulk flow sorting was performed using a BD FACSAria™ III cell sorter at the icddr,b. Bulk sorted plasmablasts were utilized immediately in ELISPOT assays as described below. Single cells were sorted into microtiter plates containing a hypotonic catch buffer and RNAse-inhibitor as described previously (4, 5). Sorted single cells were stored at -80ºC until use.

Antigens. LPS was prepared from *V. cholerae* O1, Ogawa (strain X-25049) or Inaba (strain T-19479), by hot phenol/water extraction followed by enzymatic treatment (DNase, RNase and protease), ultracentrifugation and dialysis against distilled water as previously described (6). OSP was purified and conjugated to bovine serum albumin (BSA) as previously described (6-8). CT was produced from a recombinant construct wherein the CT gene was cloned from *V. cholerae* 569B and expressed in *E. coli* JM83 in a pUC18 cloning vector. LT and LT-B were produced from recombinant constructs wherein the LT and LT-B genes were cloned from *E. coli* H10407 and expressed in *E. coli* JM83 in pUC18 cloning vectors. Recombinant proteins were purified by galactose affinity chromatography as previously described (9). Recombinant TcpA was produced and purified as previously described (10). Sodium azide-free *V. cholerae* CT (List Biologicals Laboratories), purified recombinant CtxB (Reagent Proteins) and purified *V. cholerae* sialidase (Roche) were purchased from commercial vendors.

ELISPOT assay. To enumerate total and CT-specific IgG ASCs, we used a previously described enzyme-linked immunosorbent spot (ELISPOT) procedure (3). Nitrocellulose-bottom plates were coated with GM1 ganglioside (3 nM/ml), or affinity-purified goat anti-human Ig (total Ig) (Jackson Immunology Research) (5 µg/ml). Plates were incubated overnight at 4°C. On the next day, recombinant CtxB was applied to the GM1-coated plates. All plates were then blocked with RPMI 1640 containing 10% fetal bovine serum. Bulk sorted plasmablasts were added directly to the plates and were incubated for 3 hours at 37°C with 5% CO_2. Horseradish peroxidase-conjugated mouse anti-human IgG antibodies (Hybridoma Reagent Laboratory) were then added at a 1:500 dilution. After overnight incubation at 37°C, the plates were washed and developed with 3-amino-9-ethyl carbazole (AEC) and the number of total and CT-specific IgG ASCs were enumerated.

Monoclonal antibody generation. mAbs were generated from single-cell sorted plasmablasts by single-cell expression cloning, essentially as previously described (4, 5). Briefly, total cDNA was prepared from sorted cells by random hexamer primed reverse transcription (Sensiscript, Qiagen). Ig heavy chain and light chain (kappa/lambda) variable domain sequences were amplified by nested PCR using chain-specific primer cocktails encompassing all variable (V) gene families and the constant domain (11). Amplification of variable domains derived from IgG, IgA, and IgM ASCs was performed by multiplexing the constant domain specific

antisense primers (11). Sense primers used in the second round of nested PCR were modified by fusing the 5' end of each primer to the M13R sequence (5'-AACAGCTATGACCATG-3'). PCR reactions were performed using Hot Start Taq Plus Master Mix (Qiagen) and primers were used at a final concentration of 200 nM per primer. Purified nested PCR products were sequenced in order to identify the V and J gene rearrangement of the heavy and light chain. Afterwards, a second PCR was performed using a high-fidelity DNA polymerase (Phusion Hot Start II, NEB) and V and J gene family specific primers that incorporated Ig chain specific restriction sites. Variable domains were then directionally cloned into human mAb heavy chain (IgG1) and light chain (kappa/lambda) expression vectors (Genbank accession numbers FJ475055, FJ475056, and FJ517647). Variable domains containing internal restriction sites were amplified by a modified PCR containing 50 µM 5-methyl-dCTP (NEB) and 150 µM dCTP. This modification did not affect PCR fidelity and abrogated the truncation of variable domains after restriction digestion. Following vector construction and sequence confirmation, heavy and light chain vectors were transiently co-transfected into Expi293F cells according manufacturer's instructions (Life Technologies). Antibodies were purified from cell culture supernatants using protein-A conjugated agarose beads (Pierce).

Sequence analyses. Germline variable domain gene usage and somatic hypermutation was analyzed using the international ImMunoGeneTics (IMGT) online information system (12) and the NCBI web based tool IgBLAST (13). Somatic hypermutation levels in V_H genes represent the number of nucleotide substitutions in FR1 through CDR3 relative to the closest germline sequence match in the IMGT database (accessed November, 2015). Plasmablast clonal expansions were determined by multiple sequence alignments of heavy and light chains with matching Ig gene rearrangements. Cells with identical junctional diversity in both the heavy and light chain were grouped as part of a single clonal expansion.

ELISA assays. All antigens were coated overnight at room temperature on Nunc MaxiSorp plates in 50 mM carbonate-bicarbonate buffer pH 9.6. The recombinant protein antigens CT, CtxB, LT, LtxB, as well as BSA-conjugated O-antigen specific polysaccharide (OSP) were coated at a concentration of 1 µg/ml. Purified LPS was coated at a concentration of 25 µg/ml. Plates were blocked for two hours at 37°C in PBS-1% BSA. Ten 1:3 serial dilutions of mAbs were evaluated at a starting concentration of 1 µg/ml. To detect bound IgG,

horseradish peroxidase-conjugated goat anti-human IgG (Jackson ImmunoResearch) was diluted at 1:5000 and incubated for 90 minutes at 37°C. Intermediate washes consisted of PBS-0.05% Tween (3X) and PBS (1X). Development was performed using 1 mg/ml o-phenylenediamine (OPD) in 0.05 M phosphate-citrate buffer pH 5.0, supplemented with 0.012% hydrogen peroxide before use. Reactions were stopped with 1 M HCl and absorbance was measured at 490 nM.

***V. cholerae* protein microarray analysis.** *V. cholerae* proteome microarrays were produced using genomic DNA from the O1 El Tor reference strain N16961 as template. ORFs larger than 150 base-pairs were amplified by PCR and cloned into pXi expression vectors (14). Genes larger than 3 kb were cloned in multiple segments as previously described (14). Cloning success was monitored by agarose gel electrophoresis to confirm PCR product insertion into the cloning vector. As an additional quality control, 20% of the plasmids of variable insert size were selected randomly and sequenced to verify target sequence match. Plasmids were expressed by *in vitro* transcription/translation (IVTT) system using RTS 100 *E. coli* HY kit (Biotech Rabbit) according to the manufacturer's protocol. Expressed proteins were printed using Omni Grid 100 microarray printer (Genomic Solutions) onto nitrocellulose-coated glass slides (Grace Bio-Labs). In addition to IVTT expressed proteins, each chip contained control spots of IVTT reactions without the addition of a plasmid ('no-DNA' control). Other control spots included purified IgGs, and purified cholera antigens including recombinant-CtxB, CT, LPS and OSP as described above. Synthesized proteins had a 10X histidine tag at N-terminus. A total of 3,647 proteins were spotted on the array. Protein expression was confirmed by probing arrays with anti-histidine antibody followed by a biotinylated anti-mouse secondary antibody (Jackson ImmunoReseach) and streptavidin-conjugated SureLight® P-3 (Columbia Biosciences). Protein expression was considered positive if signal intensity by anti-histidine antibody was above the mean of 'no-DNA' controls (IVTT without the addition of a plasmid) plus 2 SD. Overall protein expression was 98.9%.

For initial probing, all monoclonal antibodies of unknown specificity were combined into pools of nine by diluting the individual antibodies to a concentration of 1 ng/µl in Protein Array Blocking Buffer (GVS). The resulting pools were spiked with a known anti-CtxB and anti-OSP antibody controls at concentrations of 0.1 ng/µl. The pooled samples were then applied to the array and incubated overnight at 4ºC. After washing in TBS-T (Tris buffered saline-Tween-20; ChemCruz), microarrays were developed using biotinylated Mouse anti-

Human IgG1 Fc secondary antibody (Thermo Fisher Scientific) followed by streptavidin-conjugated SureLight® P-3 (Columbia Biosciences) as described previously (15). After an initial analysis of proteins reactive with the pooled antibodies as well as cholera patient plasma (data not shown), a targeted microarray (mini-array) was fabricated containing the top 100 cholera antigenic hits. These microarrays were then used to screen individual monoclonal antibodies. Each mAb was probed at a concentration of 2 µg/ml as described above.

The array signal intensities were quantified using Pro Scan Array Express software (Perkin Elmer). Mean pixel intensities were corrected for spot-specific background. Antibody-antigen pairs were considered positive if they gave a signal greater than 10 times the array signal intensity range of an isotype control antibody (EM4C04, influenza HA specific IgG1). This threshold was chosen to maximize confidence in the identified pairs, but may have missed some very low affinity interactions, or proteins that were poorly expressed and/or folded. For each of these antibodies for which we could identify a target antigen, different concentrations of individual mAbs ranging between 0.001-100 µg/ml (depending on signal intensity obtained for each antibody at initial screening) were titrated on the *V. cholerae* proteome microarray, and the binding curves of *V. cholerae*-specific mAbs were generated from two measurements.

Toxin neutralization assays. CT and LT neutralization assays were performed using Caco-2 cells grown to confluency in 96-well tissue culture plates. Two-fold dilutions of each mAb were performed in DMEM starting at 40 µg/ml. Diluted antibodies were mixed with an equal volume of DMEM containing *V. cholerae* CT (List Biologicals) or trypsin-activated LT to achieve a final toxin concentration of 16.6 ng/ml. Mixtures were incubated at RT for 2 hours. For activation of LT before combination with mAbs, 10 ng of trypsin (Sigma, T8003) suspended in water was added to 10 µl of recombinant LT at 1 mg/ml and incubated for 1 hour at 37°C. Cells were gently washed with PBS and media was replaced with 70 µL of DMEM before adding 30 µL of each mAb-CT mixture to the cells, which were then incubated for 3 hours at 37°C in 5% CO_2. Media was replaced with 130 µL of 0.1 M HCl-0.1% Triton-X cell lysis solution, and cells were lysed for 10 minutes at 4°C. Cell lysate supernatants were collected after centrifugation (660 x g, 10 min). Cyclic AMP levels were measured in 100 µl of cell lysates using a commercial Direct cAMP ELISA kit (Enzo Life Sciences). EC_{50} values were determined as the concentration of mAb that effected a 50% reduction in intracellular cAMP levels relative to a media only control.

Sialidase activity assay. For measuring the potentiating effect of sialidase on CT toxicity, and how the sialidase-specific mAbs modulated this, we seeded 96-well plates with Caco-2 cells using methods identical to toxin neutralization assays. Monoclonal antibodies were serially diluted 1:9 in PBS starting at 13.3 µg/ml. Antibodies were mixed with an equal volume of 2 mU/ml *V. cholerae* sialidase (Roche) in DMEM and incubated for 1 hour at RT. 70 µl of sialidase or sialidase with antibody was added to cells washed with PBS and incubated for 2 hours at 37°C with 5% CO_2. 30 µl of *V. cholerae* CT (List Biological Laboratories) at 1.67 pg/µl in DMEM was added to wells and cells were incubated for an additional 3 hours. Cell lysates were collected and cAMP levels were measured as described above.

Vibriocidal assays. Assays were performed using *V. cholerae* strains O1-Ogawa (X25049) and O1-Inaba (T19479). Vibriocidal assays were essentially conducted as previously described (16). In this study, mAbs were diluted two-fold in 0.9% saline solution starting at 10 µg/ml. Vibriocidal EC_{50} values were determined as the concentration of mAb that effected a 50% reduction in the average culture turbidity (OD 600) of media-only controls.

Bacterial agglutination. To determine minimum agglutinating titers, antibodies were diluted two-fold in PBS starting at a concentration of 20 µg/ml. Bacteria were prepared by growing each *V. cholerae* strain O1-Ogawa (X25049) and O1-Inaba (T19479) to mid-log phase (2-3 hours) in bovine heart infusion media. Bacteria were then pelleted at 3,000 x g for 10 min and washed twice in PBS. Immediately before use, bacteria were normalized such that a 1:10 dilution of the sample had an OD 600 value of 0.4 ± 0.02 relative to PBS. For each antibody, duplicate measurements were performed in which 25 µL of the bacterial sample was combined with an equal volume of diluted mAbs added to a V-bottom microtiter plate. Plates were sealed with adhesive film, briefly centrifuged to concentrate contents at the bottom of the well (20 x g, 15 seconds), mixed, and then incubated for 20-24 hours at 4°C to allow non-agglutinated bacteria to form a ‘button’ at the bottom of the well. Plates were imaged using a UV imaging system (ChemiDoc, BioRad). Agglutination titers were recorded as the last dilution where all bacteria were visibly agglutinated.

2. Supplemental Figures and Tables

Figure S1. *V. cholerae* infection results in a potent and specific plasmablast response that has undergone isotype switching, somatic hypermutation, and clonal expansion. **(A)** The immunoglobulin isotype frequency of single cells as determined by sequencing. Numbers in parentheses represent the total number of single cells analyzed. **(B)** The mean number of somatic mutations in the V_H gene for each study participant is shown; bars represent median values. Published historical results from previous studies of influenza and dengue fever are provided for comparison (17, 18). **(C)** The percentage of Ig sequences from each patient that were derived from clonally related cells, as indicated by shared V_H and J_H segments, as well as CDR3 junctional diversity are shown, with comparisons to previously published data on systemic viral infections; bars represent median values (17, 18).

Figure S2. Cholera toxin-specific antibodies. Antibody affinity to cholera holotoxin and CtxB. Antibodies denoted by red bars bound to CT holotoxin but not the CtxB subunit at a concentration of 1 µg/ml (dotted line). Each antibody was measured in at least two independent experiments.

Figure S3. *V. cholerae*-specific mAbs show a dose dependent binding to four antigens using protein microarray. Binding curves of sialidase, FlaA-, TagA-, and phosphocarrier protein kinase-specific mAbs were generated using the targeted array. Different concentrations of antibody ranging from 100 to 0.001 µg/ml were titrated on microarray chips. For all antibodies, the signal intensity was antibody concentration dependent.

Figure S4. Expansion of highly mutated LPS specific antibodies that preferentially recognize a currently non-circulating *V. cholerae* strain. **(A)** For three study participants, the plasmablasts derived from clonally expanded populations are grouped and shown as expanded sections in the pie charts. Clonal expansions (CE) containing LPS-specific antibodies are shown in red and numbered to correspond to specific comparisons between clonal expansions described below. **(B)** Antibody affinity by ELISA to LPS is shown as the minimum mAb concentration required for three times the background signal of sample dilution buffer. **(C)** EC_{50} values corresponding to the vibriocidal activity of each mAb are shown. In panels B and C, mAbs

belonging to a unique clonal expansion (CE) are grouped. The asterisk denotes antibody CF21.2.F1. **(D)** V_H mutations observed for CT and LPS-specific antibodies. Clonal expansions for LPS-specific antibodies are denoted as follows: Group 1: circles, group 2: squares, group 3: triangles. **(E)** Phylogram generated by maximum likelihood analysis of heavy chain variable domain sequences derived from the highly mutated LPS-specific clonal expansion #1 observed in patient CF21.

Figure S5. Comparative analysis of vibriocidal and agglutination functional characteristics. (A & B) Strains and LPS used in these assays were derived from *V. cholerae* O1-Ogawa (left) and O1-Inaba (right). Lines represent linear regression analysis of $\log_{10}$ transformed values. Outliers that were below the limit of detection in both assays were excluded from regression analyses. **(A)** Correlation between vibriocidal EC_{50} values (y-axis) and the minimum positive binding concentration in an ELISA (x-axis). **(B)** Correlation between the minimum agglutination antibody concentration (y-axis) and the minimum positive binding concentration in an ELISA (x-axis). **(C)** Representative analysis of antibody-mediated vibriocidal activity is shown to *V. cholerae* O1-Ogawa for mAb AT11.1.A04. Bars show SEM of the assay measured in triplicate. Higher values on the y-axis correspond to increased culture turbidity as measured by UV absorbance at 600 nM. EC_{50} values were determined as the concentration of mAb that effected a 50% reduction in bacterial growth (dotted line). **(D)** Representative analysis of antibody-mediated bacterial agglutination with 2 fold titrated is mAb shown. White arrows depict the well displaying the minimal agglutination concentration.

Table SI. Cholera patient cohort data

Table SII. Summary of monoclonal antibody panel

Table SIII. Summary of mAb specificity

Table SIV: Summary of monoclonal antibody characteristics

3. Supplemental References

1. **Qadri, F., T. R. Bhuiyan, K. K. Dutta, R. Raqib, M. S. Alam, N. H. Alam, A. M. Svennerholm, and M. M. Mathan.** 2004. Acute dehydrating disease caused by Vibrio cholerae serogroups O1 and O139 induce increases in innate cells and inflammatory mediators at the mucosal surface of the gut. Gut. **53:**62-69.

2. **Qadri, F., S. K. Das, A. S. Faruque, G. J. Fuchs, M. J. Albert, R. B. Sack, and A. M. Svennerholm.** 2000. Prevalence of toxin types and colonization factors in enterotoxigenic Escherichia coli isolated during a 2-year period from diarrheal patients in Bangladesh. J. Clin. Microbiol. **38:**27-31.

3. **Rahman, A., R. Rashu, T. R. Bhuiyan, F. Chowdhury, A. I. Khan, K. Islam, R. C. LaRocque, E. T. Ryan, J. Wrammert, S. B. Calderwood, F. Qadri, and J. B. Harris.** 2013. Antibody-secreting cell responses after Vibrio cholerae O1 infection and oral cholera vaccination in adults in Bangladesh. Clin. Vaccine Immunol. **20:**1592-1598. doi: 10.1128/CVI.00347-13 [doi].

4. **Wrammert, J., K. Smith, J. Miller, W. A. Langley, K. Kokko, C. Larsen, N. Y. Zheng, I. Mays, L. Garman, C. Helms, J. James, G. M. Air, J. D. Capra, R. Ahmed, and P. C. Wilson.** 2008. Rapid cloning of high-affinity human monoclonal antibodies against influenza virus. Nature. **453:**667-671. doi: 10.1038/nature06890.

5. **Smith, K., L. Garman, J. Wrammert, N. Y. Zheng, J. D. Capra, R. Ahmed, and P. C. Wilson.** 2009. Rapid generation of fully human monoclonal antibodies specific to a vaccinating antigen. Nat. Protoc. **4:**372-384. doi: 10.1038/nprot.2009.3.

6. **Xu, P., M. M. Alam, A. Kalsy, R. C. Charles, S. B. Calderwood, F. Qadri, E. T. Ryan, and P. Kovac.** 2011. Simple, direct conjugation of bacterial O-SP-core antigens to proteins: development of cholera conjugate vaccines. Bioconjug. Chem. **22:**2179-2185. doi: 10.1021/bc2001984.

7. **Sayeed, M. A., M. K. Bufano, P. Xu, G. Eckhoff, R. C. Charles, M. M. Alam, T. Sultana, M. R. Rashu, A. Berger, G. Gonzalez-Escobedo, A. Mandlik, T. R. Bhuiyan, D. T. Leung, R. C. LaRocque, J. B. Harris, S. B. Calderwood, F. Qadri, W. F. Vann, P. Kovac, and E. T. Ryan.** 2015. A Cholera Conjugate Vaccine Containing O-specific Polysaccharide (OSP) of V. cholerae O1 Inaba and Recombinant Fragment of Tetanus Toxin Heavy Chain (OSP:rTTHc) Induces Serum, Memory and Lamina Proprial Responses against OSP and Is Protective in Mice. PLoS Negl Trop. Dis. **9:**e0003881. doi: 10.1371/journal.pntd.0003881 [doi].

8. **Uddin, T., A. Aktar, P. Xu, R. A. Johnson, M. A. Rahman, D. T. Leung, S. Afrin, A. Akter, M. M. Alam, A. Rahman, F. Chowdhury, A. I. Khan, T. R. Bhuiyan, M. K. Bufano, R. Rashu, Y. Yu, Y. Wu-Freeman, J. B. Harris, R. C. LaRocque, R. C. Charles, P. Kovac, S. B. Calderwood, E. T. Ryan, and F. Qadri.** 2014. Immune responses to O-specific polysaccharide and lipopolysaccharide of Vibrio cholerae O1 Ogawa in adult Bangladeshi recipients of an oral killed cholera vaccine and comparison to responses in patients with cholera. Am. J. Trop. Med. Hyg. **90:**873-881. doi: 10.4269/ajtmh.13-0498 [doi].

9. **Norton, E. B., L. B. Lawson, L. C. Freytag, and J. D. Clements.** 2011. Characterization of a mutant Escherichia coli heat-labile toxin, LT(R192G/L211A), as a safe and effective oral adjuvant. Clin. Vaccine Immunol. **18:**546-551. doi: 10.1128/CVI.00538-10.

10. **Rollenhagen, J. E., A. Kalsy, F. Cerda, M. John, J. B. Harris, R. C. Larocque, F. Qadri, S. B. Calderwood, R. K. Taylor, and E. T. Ryan.** 2006. Transcutaneous immunization with toxin-coregulated pilin A induces protective immunity against Vibrio cholerae O1 El Tor challenge in mice. Infect. Immun. **74:**5834-5839. doi: 74/10/5834 [pii]; 10.1128/IAI.00438-06 [doi].

11. **Tiller, T., E. Meffre, S. Yurasov, M. Tsuiji, M. C. Nussenzweig, and H. Wardemann.** 2008. Efficient generation of monoclonal antibodies from single human B cells by single cell RT-PCR and expression vector cloning. J. Immunol. Methods. **329:**112-124. doi: S0022-1759(07)00312-2 [pii].

12. **Lefranc, M. P., V. Giudicelli, Q. Kaas, E. Duprat, J. Jabado-Michaloud, D. Scaviner, C. Ginestoux, O. Clement, D. Chaume, and G. Lefranc.** 2005. IMGT, the international ImMunoGeneTics information system. Nucleic Acids Res. **33:**D593-7. doi: 33/suppl_1/D593 [pii].

13. **Ye, J., N. Ma, T. L. Madden, and J. M. Ostell.** 2013. IgBLAST: an immunoglobulin variable domain sequence analysis tool. Nucleic Acids Res. **41:**W34-40. doi: 10.1093/nar/gkt382 [doi].

14. **Davies, D. H., X. Liang, J. E. Hernandez, A. Randall, S. Hirst, Y. Mu, K. M. Romero, T. T. Nguyen, M. Kalantari-Dehaghi, S. Crotty, P. Baldi, L. P. Villarreal, and P. L. Felgner.** 2005. Profiling the humoral immune response to infection by using proteome microarrays: high-throughput vaccine and diagnostic antigen discovery. Proc. Natl. Acad. Sci. U. S. A. **102:**547-552. doi: 0408782102 [pii]; 10.1073/pnas.0408782102 [doi].

15. **Vigil, A., C. Chen, A. Jain, R. Nakajima-Sasaki, A. Jasinskas, J. Pablo, L. R. Hendrix, J. E. Samuel, and P. L. Felgner.** 2011. Profiling the humoral immune response of acute and chronic Q fever by protein microarray. Mol. Cell. Proteomics. **10:**M110.006304. doi: 10.1074/mcp.M110.006304 [doi].

16. **Harris, A. M., M. S. Bhuiyan, F. Chowdhury, A. I. Khan, A. Hossain, E. A. Kendall, A. Rahman, R. C. LaRocque, J. Wrammert, E. T. Ryan, F. Qadri, S. B. Calderwood, and J. B. Harris.** 2009. Antigen-specific memory B-cell responses to Vibrio cholerae O1 infection in Bangladesh. Infect. Immun. **77:**3850-3856. doi: 10.1128/IAI.00369-09.

17. **Wrammert, J., D. Koutsonanos, G. M. Li, S. Edupuganti, J. Sui, M. Morrissey, M. McCausland, I. Skountzou, M. Hornig, W. I. Lipkin, A. Mehta, B. Razavi, C. Del Rio, N. Y. Zheng, J. H. Lee, M. Huang, Z. Ali, K. Kaur, S. Andrews, R. R. Amara, Y. Wang, S. R. Das, C. D. O'Donnell, J. W. Yewdell, K. Subbarao, W. A. Marasco, M. J. Mulligan, R. Compans, R. Ahmed, and P. C. Wilson.** 2011. Broadly cross-reactive antibodies dominate the human B cell response against 2009 pandemic H1N1 influenza virus infection. J. Exp. Med. **208:**181-193. doi: 10.1084/jem.20101352.

18. **Priyamvada, L., A. Cho, N. Onlamoon, N. Y. Zheng, M. Huang, Y. Kovalenkov, K. Chokephaibulkit, N. Angkasekwinai, K. Pattanapanyasat, R. Ahmed, P. C. Wilson, and J. Wrammert.** 2016. B Cell Responses during Secondary Dengue Virus Infection Are Dominated by Highly Cross-Reactive, Memory-Derived Plasmablasts. J. Virol. **90:**5574-5585. doi: 10.1128/JVI.03203-15 [doi].
