# Supplementary material for: Single-Cell Analysis of the Plasmablast Response to Vibrio cholerae Demonstrates Expansion of Cross-Reactive Memory B Cells
Source: mBio. 2016 Dec 20;7(6):e02021-16. doi: 10.1128/mBio.02021-16 (PMC5181778; doi:10.1128/mBio.02021-16)
Supplement: Figure S1 — V. cholerae infection results in a potent and specific plasmablast response that has undergone isotype switching, somatic hypermutation, and clonal expansion. (A) The immunoglobulin isotype frequency of single cells as determined by sequencing. Numbers in parentheses represent the total number of single cells analyzed. (B) The mean number of somatic mutations in the VH gene for each study participant is shown; bars represent median values. Published historical results from previous studies of influenza and dengue fever are provided for comparison (14, 16). (C) The percentages of Ig sequences from each patient that were derived from clonally related cells, as indicated by shared VH and JH segments, as well as CDR3 junctional diversity are shown, with comparisons to previously published data on systemic viral infections; bars represent median values (14, 16). Download [file mbo006163110sf1.pdf]

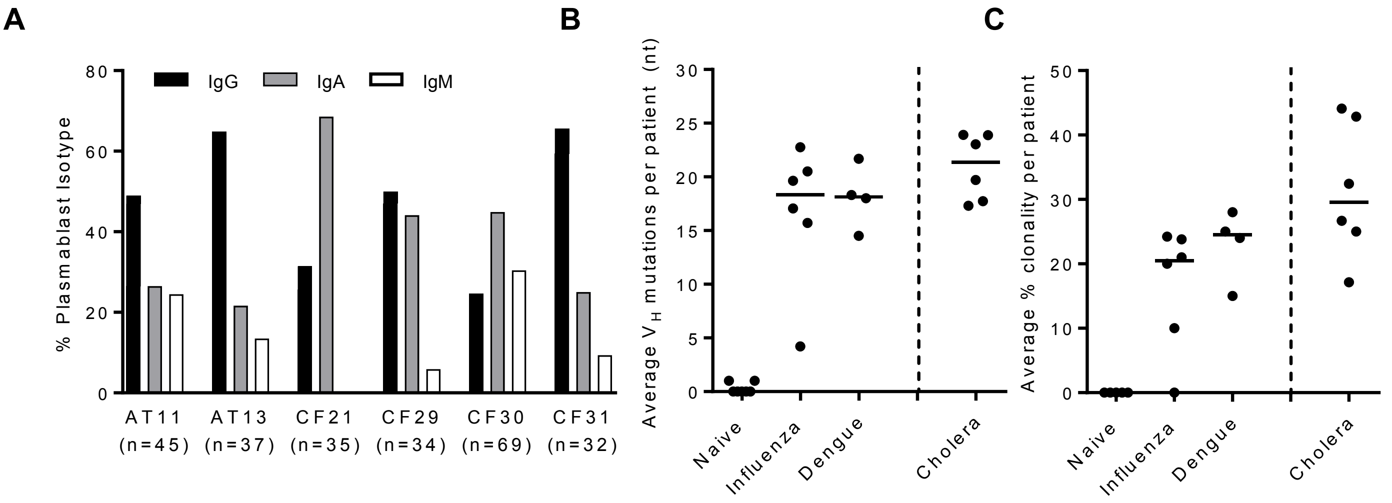

Figure S1. ***V. cholerae*** **infection results in a potent and specific plasmablast response that has undergone isotype switching, somatic hypermutation, and clonal expansion. (A)** The immunoglobulin isotype frequency of single cells as determined by sequencing. Numbers in parentheses represent the total number of single cells analyzed. **(B)** The mean number of somatic mutations in the V_H gene for each study participant is shown; bars represent median values. Published historical results from previous studies of influenza and dengue fever are provided for comparison (Wrammert et al., 2011, Priyamvada et al., 2016). **(C)** The percentage of Ig sequences from each patient that were derived from clonally related cells, as indicated by shared V_H and J_H segments, as well as CDR3 junctional diversity are shown, with comparisons to previously published data on systemic viral infections; bars represent median values (Wrammert et al., 2011, Priyamvada et al., 2016).
