# Supplementary material for: Single-Cell Analysis of the Plasmablast Response to Vibrio cholerae Demonstrates Expansion of Cross-Reactive Memory B Cells
Source: mBio. 2016 Dec 20;7(6):e02021-16. doi: 10.1128/mBio.02021-16 (PMC5181778; doi:10.1128/mBio.02021-16)
Supplement: Figure S2 — Cholera toxin-specific antibodies. The antibody affinity to cholera holotoxin and CtxB is shown. Antibodies denoted by red bars bound to CT holotoxin but not the CtxB subunit at a concentration of 1 µg/ml (dotted line). Each antibody was measured in at least two independent experiments. Download [file mbo006163110sf2.pdf]

Figure S2

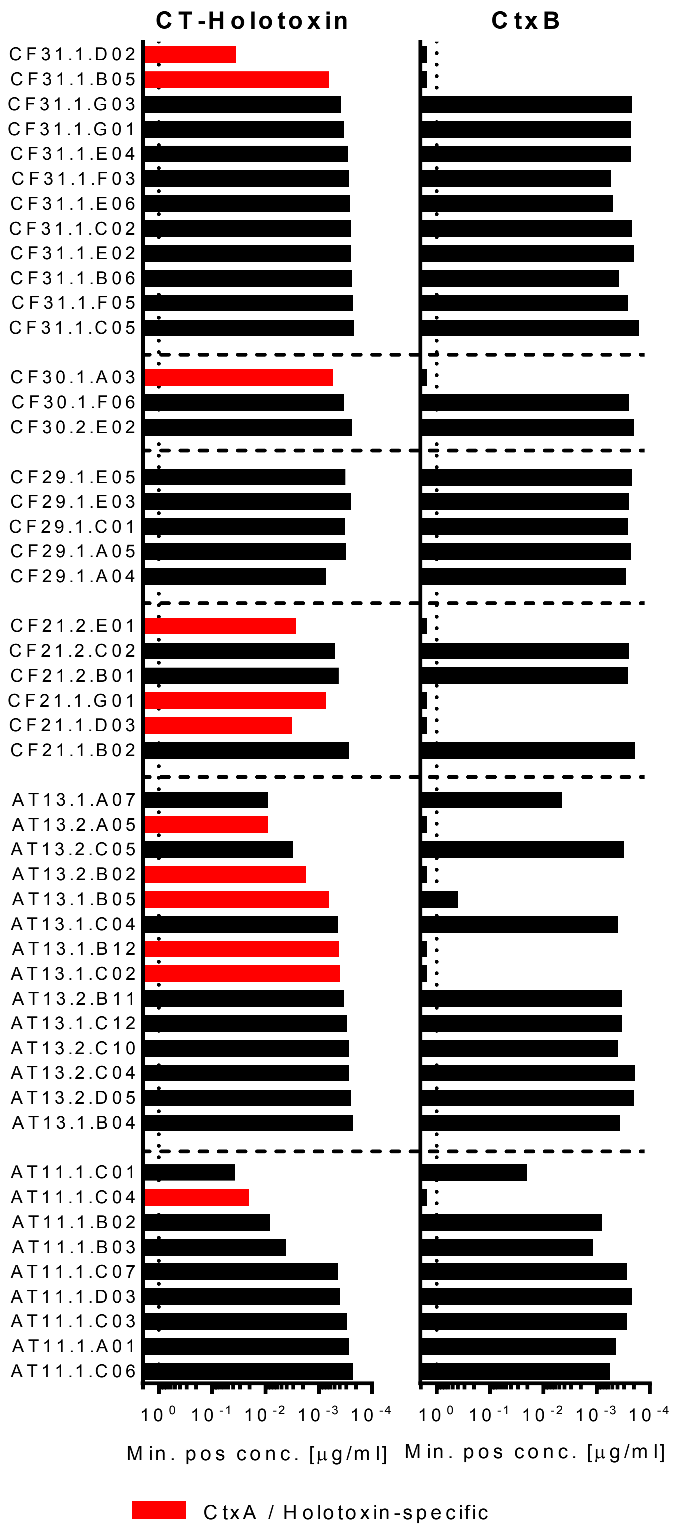

Figure S2. Cholera toxin-specific antibodies. Antibody affinity to cholera holotoxin and CtxB. Antibodies denoted by red bars bound to CT holotoxin but not the CtxB subunit at a concentration of 1 µg/ml (dotted line). Each antibody was measured in at least two independent experiments.
