# Supplementary material for: Single-Cell Analysis of the Plasmablast Response to Vibrio cholerae Demonstrates Expansion of Cross-Reactive Memory B Cells
Source: mBio. 2016 Dec 20;7(6):e02021-16. doi: 10.1128/mBio.02021-16 (PMC5181778; doi:10.1128/mBio.02021-16)
Supplement: Figure S3 — V. cholerae-specific MAbs show a dose-dependent binding to four antigens using a protein microarray. Binding curves of sialidase, FlaA-, TagA-, and phospho-carrier protein kinase-specific MAbs were generated using the targeted array. Different concentrations of antibody ranging from 100 to 0.001 µg/ml were titrated on microarray chips. For all antibodies, the signal intensity was antibody concentration dependent. Download [file mbo006163110sf3.pdf]

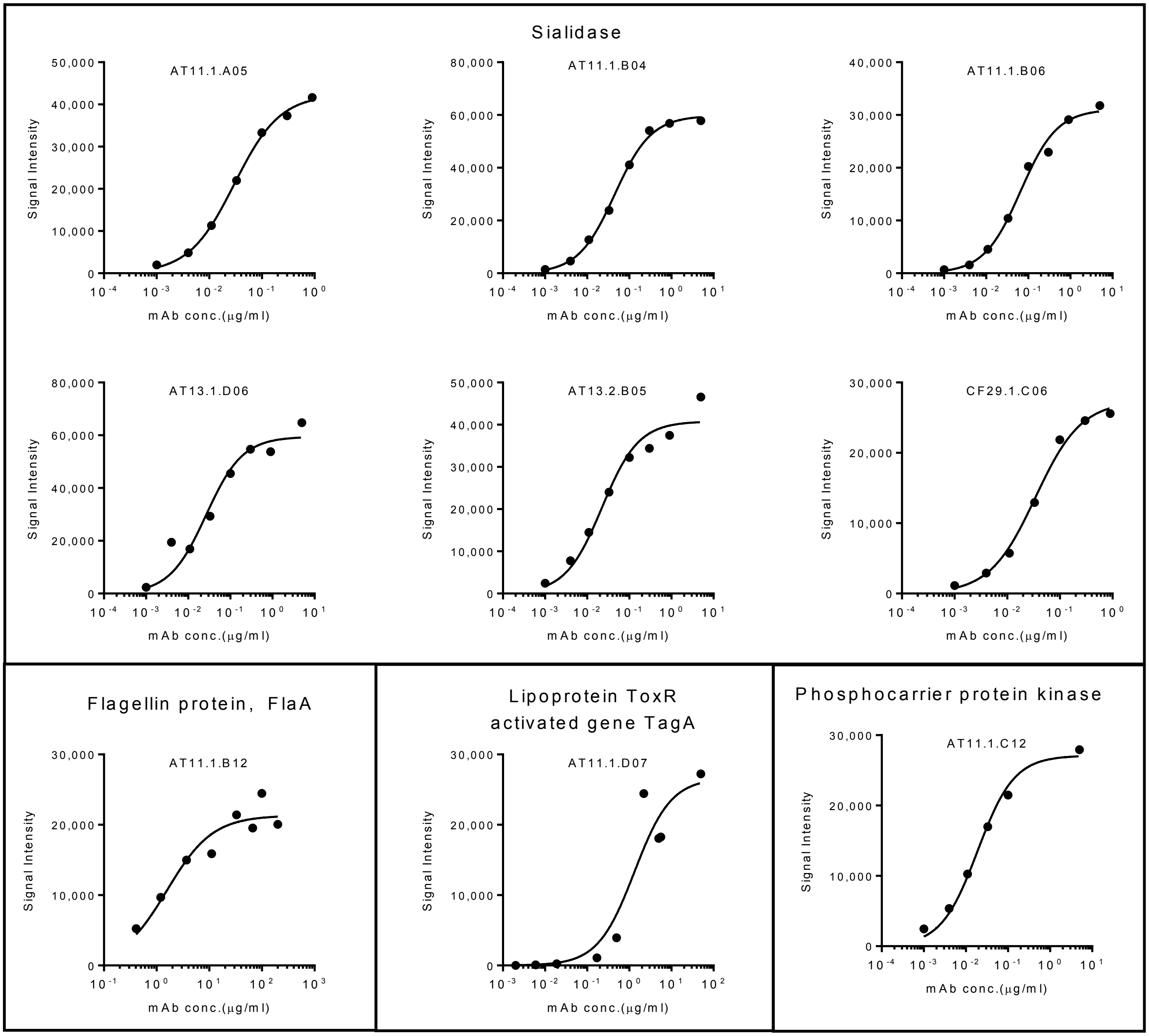

Figure S3. *V. cholerae*-specific mAbs show a dose dependent binding to four antigens using protein microarray. Binding curves of sialidase, FlaA-, TagA-, and phosphocarrier protein kinase-specific mAbs were generated using the targeted array. Different concentrations of antibody ranging from 100 to 0.001 µg/ml were titrated on microarray chips. For all antibodies, the signal intensity was antibody concentration dependent.
