# Supplementary material for: Single-Cell Analysis of the Plasmablast Response to Vibrio cholerae Demonstrates Expansion of Cross-Reactive Memory B Cells
Source: mBio. 2016 Dec 20;7(6):e02021-16. doi: 10.1128/mBio.02021-16 (PMC5181778; doi:10.1128/mBio.02021-16)
Supplement: Figure S4 — Expansion of highly mutated LPS-specific antibodies that preferentially recognize a currently noncirculating V. cholerae strain. (A) For three study participants, the plasmablasts derived from clonally expanded populations are grouped and shown as expanded sections in the pie charts. Clonal expansions (CE) containing LPS-specific antibodies are shown in red and numbered to correspond to specific comparisons between clonal expansions described below. (B) Antibody affinity by ELISA to LPS is shown as the minimum MAb concentration required for 3 times the background signal of sample dilution buffer. (C) EC50s corresponding to the vibriocidal activity of each MAb are shown. In panels B and C, MAbs belonging to a unique clonal expansion are grouped. The asterisk denotes antibody CF21.2.F1. (D) VH mutations observed for CT- and LPS-specific antibodies. Clonal expansions for LPS-specific antibodies are denoted as follows: group 1, circles; group 2, squares; group 3, triangles. (E) Phylogram generated by maximum likelihood analysis of heavy-chain variable domain sequences derived from the highly mutated LPS-specific clonal expansion no. 1 observed in patient CF21. Download [file mbo006163110sf4.pdf]

Figure S4

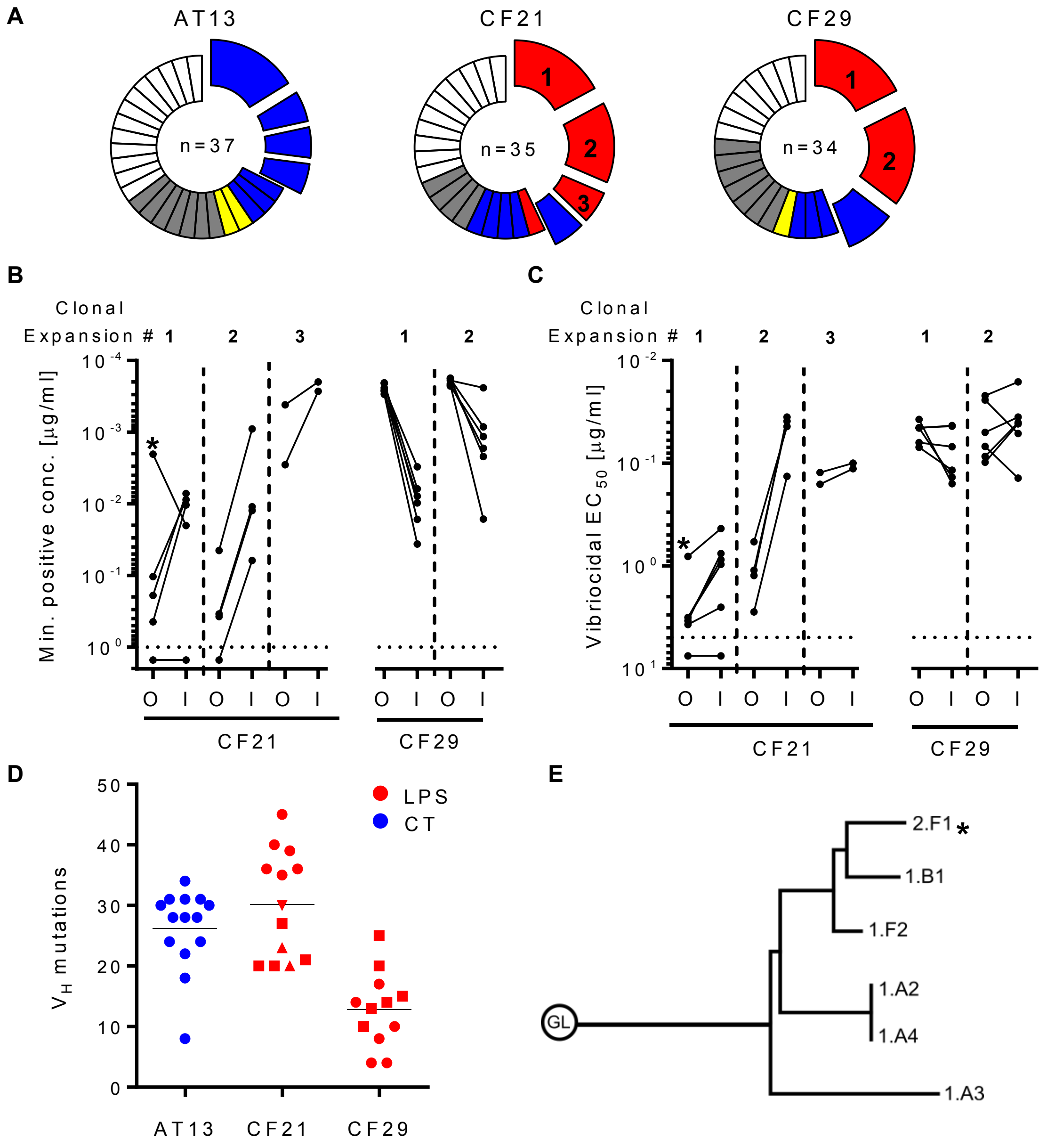

Figure S4. Expansion of highly mutated LPS specific antibodies that preferentially recognize a currently non-circulating *V. cholerae* strain. (A) For three study participants, the plasmablasts derived from clonally expanded populations are grouped and shown as expanded sections in the pie charts. Clonal expansions (CE) containing LPS-specific antibodies are shown in red and numbered to correspond to specific comparisons between clonal expansions described below. **(B)** Antibody affinity by ELISA to LPS is shown as the minimum mAb concentration required for three times the background signal of sample dilution buffer. **(C)** EC_{50} values corresponding to the vibriocidal activity of each mAb are shown. In panels B and C, mAbs belonging to a unique clonal expansion (CE) are grouped. The asterisk denotes antibody CF21.2.F1. **(D)** V_H mutations observed for CT and LPS-specific antibodies. Clonal expansions for LPS-specific antibodies are denoted as follows: Group 1: circles, group 2: squares, group 3: triangles. **(E)** Phylogram generated by maximum likelihood analysis of heavy chain variable domain sequences derived from the highly mutated LPS-specific clonal expansion #1 observed in patient CF21.
