# Supplementary material for: Single-Cell Analysis of the Plasmablast Response to Vibrio cholerae Demonstrates Expansion of Cross-Reactive Memory B Cells
Source: mBio. 2016 Dec 20;7(6):e02021-16. doi: 10.1128/mBio.02021-16 (PMC5181778; doi:10.1128/mBio.02021-16)
Supplement: Figure S5 — Comparative analysis of vibriocidal and agglutination functional characteristics (A and B). The strains and LPS used in these assays were derived from V. cholerae O1-Ogawa (left) and O1-Inaba (right). Lines represent linear regression analysis of log10-transformed values. Outliers that were below the limit of detection in both assays were excluded from regression analyses. (A) Correlation between vibriocidal EC50 values (y axis) and the minimum positive binding concentration in an ELISA (x axis). (B) Correlation between the minimum agglutination antibody concentration (y axis) and the minimum positive binding concentration in an ELISA (x axis). (C) Representative analysis of antibody-mediated vibriocidal activity is shown to V. cholerae O1-Ogawa for MAb AT11.1.A04. Bars show SEM of the assay measured in triplicate. Higher values on the y axis correspond to increased culture turbidity as measured by UV absorbance at 600 nm. EC50s were determined as the concentration of MAb that effected a 50% reduction in bacterial growth (dotted line). (D) Representative analysis of antibody-mediated bacterial agglutination with 2-fold titration of the MAb shown. White arrows depict the well displaying the minimal agglutination concentration. Download [file mbo006163110sf5.pdf]

Figure S5

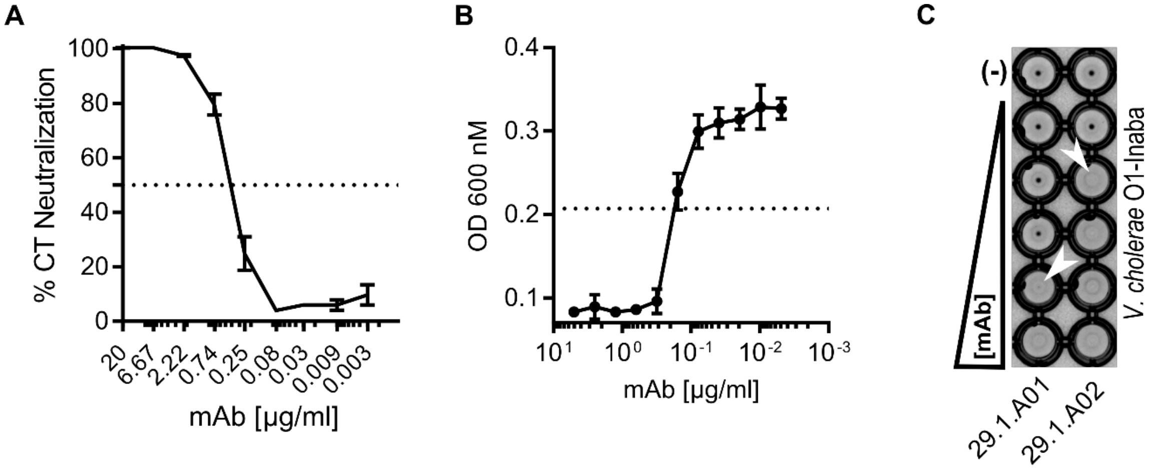

Figure S5. Representative functional assay data. (A) Analysis of antibody-mediated toxin neutralization (mAb: CF29.1.E03). Values show the mean percent reduction in intracellular cAMP levels relative to a no-antibody treatment control. Bars represent the standard deviation of two technical replicates. The dotted line indicates the 50% reduction in CT-induced cAMP levels relative to a no-antibody control. This threshold was used to calculate toxin neutralization EC_{50} values for each mAb; bars show SEM of the assay measured in duplicate. **(B)** Antibody-mediated vibriocidal activity is shown to *V. cholerae* O1-Ogawa for mAb AT11.1.A04. Bars show SEM of the assay measured in triplicate. Higher values on the y-axis correspond to increased culture turbidity as measured by UV absorbance at 600 nM. EC_{50} values were determined as the concentration of mAb that effected a 50% reduction in bacterial growth (dotted line). **(C)** Antibody-mediated bacterial agglutination with 2 fold titrated is mAb shown. White arrows depict the well displaying the minimal agglutination concentration.
