# Supplementary material for: Single-Cell Analysis of the Plasmablast Response to Vibrio cholerae Demonstrates Expansion of Cross-Reactive Memory B Cells
Source: mBio. 2016 Dec 20;7(6):e02021-16. doi: 10.1128/mBio.02021-16 (PMC5181778; doi:10.1128/mBio.02021-16)
Supplement: Table S1 — Cholera patient cohort data. [file mbo006163110st1.pdf]

Table SI. Cholera patient cohort data

Patient ID	Age	Gender	*V. cholerae* serogroup-type	Blood Group	% PB d7†	Single-cell PB sort	Vibriocidal titer, d2*	Vibriocidal titer, d7*
**AT11**	18	M	O1-Ogawa	O	10	Yes	5	640
**AT13**	43	F	O1-Ogawa	AB	2	Yes	40	2,560
**CF21**	20	M	O1-Ogawa	B	14	Yes	10	1,280
**CF22**	23	F	O1-Ogawa	A	1	-	-	-
**CF23**	28	M	O1-Ogawa	B	3	-	-	-
**CF24**	45	F	O1-Ogawa	O	3	-	-	-
**CF25**	35	F	O1-Ogawa	O	11	-	-	-
**CF27**	25	F	O1-Ogawa	O	9	-	-	-
**CF29**	30	M	O1-Ogawa	O	24	Yes	5	1,280
**CF30**	29	F	O1-Ogawa	O	29	Yes	5	320
**CF31**	18	F	O1-Ogawa	A	7	Yes	80	5,120

† Defined here as % CD38/CD27 hi cells of total $CD19^+$ cells
*Serum vibriocidal titers measured for *V. cholerae* O1-Ogawa
