# Supplementary material for: Single-Cell Analysis of the Plasmablast Response to Vibrio cholerae Demonstrates Expansion of Cross-Reactive Memory B Cells
Source: mBio. 2016 Dec 20;7(6):e02021-16. doi: 10.1128/mBio.02021-16 (PMC5181778; doi:10.1128/mBio.02021-16)
Supplement: Table S2 — Summary of monoclonal antibody panel. [file mbo006163110st2.pdf]

**Table SII.** Summary of monoclonal antibody panel

| Patient ID | Number $V_H$ Sequences | mAbs* IgG | IgA# | IgM# | Total |
|---|---|---|---|---|---|
| **AT11** | 49 | 17 | 5 | 4 | 26 |
| **AT13** | 37 | 20 | 1 | 0 | 21 |
| **CF21** | 35 | 10 | 12 | 0 | 22 |
| **CF29** | 34 | 17 | 7 | 0 | 24 |
| **CF30** | 69 | 16 | 5 | 4 | 25 |
| **CF31** | 32 | 19 | 0 | 1 | 20 |
| | 256 | 99 | 30 | 9 | 138 |

*All monoclonal antibodies were cloned into a human IgG1 backbone.

# IgA and IgM secreting cells were generated from clonal expansions.
