# Supplementary material for: Single-Cell Analysis of the Plasmablast Response to Vibrio cholerae Demonstrates Expansion of Cross-Reactive Memory B Cells
Source: mBio. 2016 Dec 20;7(6):e02021-16. doi: 10.1128/mBio.02021-16 (PMC5181778; doi:10.1128/mBio.02021-16)
Supplement: Table S3 — Summary of MAb specificity. [file mbo006163110st3.pdf]

Table SIII. Summary of mAb specificity

Patient	Cholera Toxin	LPS	Undetermined
**AT11**	9	1	16
**AT13**	14	0	7
**CF21**	6	11	7
**CF29**	5	12	7
**CF30**	3	0	22
**CF31**	12	0	6
**Total**	49	24	65
