# Supplementary material for: Single-Cell Analysis of the Plasmablast Response to Vibrio cholerae Demonstrates Expansion of Cross-Reactive Memory B Cells
Source: mBio. 2016 Dec 20;7(6):e02021-16. doi: 10.1128/mBio.02021-16 (PMC5181778; doi:10.1128/mBio.02021-16)
Supplement: Table S4 — Summary of monoclonal antibody characteristics. [file mbo006163110st4.pdf]

Table SIV: Summary of monoclonal antibody characteristics

Patient	Ab	Specificity (ELISA/Array)	Clonal expansion per patient	Original Ig isotype* Heavy chain	Original Ig isotype* Light chain	Rearrangement Heavy Chain V	Rearrangement Heavy Chain J	Mutations	Rearrangement Light Chain V	Rearrangement Light Chain J	Mutations
AT11	1.C04	CtxA/Holotoxin		IgG1	Igκ	3-21	3	16	4-1	1	13
	1.B03	CtxB	1	IgG1	Igκ	5-51	5	8	1-12	4	9
	1.C07	CtxB	1	IgG1	Igκ	5-51	5	10	1-12	4	13
	1.A01	CtxB	4	IgG2	Igλ	3-23	6	48	3-21	3	16
	1.C06	CtxB	4	IgG1	Igλ	3-23	6	20	3-21	2	19
	1.B02	CtxB		IgG1	Igκ	4-39	5	23	1D-33	4	13
	1.C01	CtxB		IgG1	Igλ	3-23	4	30	1-40	2	8
	1.C03	CtxB		IgG1	Igκ	5-51	5	11	1-16	4	24
	1.D03	CtxB		IgG1	Igκ	4-61	4	34	1-39	2	22
	1.B12	FlaA†	3	IgM	Igλ	4-39	5	11	1-40	2	5
	1.A04	LPS/OSP	6	IgM	Igλ	3-64	4	16	2-14	1	15
	1.D11	LPS/OSP	6	IgM	Igλ	3-64	4	10	2-18	1	6
	1.C12	PPK†		IgG1	Igλ	7-4	5	28	2-8	2	19
	1.A05	Sialidase		IgA1	Igλ	3-30	6	27	3-25	2	14
	1.B04	Sialidase		IgG1	Igκ	3-30	4	12	1-16	5	7
	1.B06	Sialidase		IgG1	Igκ	3-30	5	7	1-39	4	4
	1.D06	Sialidase		IgG1	Igλ	3-21	4	21	4-60	2	17
	1.D07	TagA†		IgG2	Igλ	4-59	5	14	1-40	2	9
	1.A02	Undetermined	2	IgA1	Igκ	5-51	5	36	4-1	5	31
	1.A06	Undetermined	2	IgA1	Igκ	5-51	4	24	4-1	5	25
	1.D01	Undetermined	3	IgM	Igλ	4-39	5	24	1-40	2	10
	1.A11	Undetermined	5	IgA1	Igκ	1-18	4	17	1-39	5	12
	1.B01	Undetermined	5	IgA1	Igκ	1-18	4	13	1-39	5	12
	1.B11	Undetermined		IgG2	Igκ	3-15	5	13	3-20	1	9
	1.C09	Undetermined		IgG2	Igκ	3-23	4	19	1-6	4	15
	1.D04	Undetermined		IgG1	Igκ	3-21	4	19	3-20	5	10
AT13	1.B05	CtxA/Holotoxin	1	IgG1	Igκ	3-21	4	24	3-20	2	19
	1.B12	CtxA/Holotoxin	1	IgG1	Igκ	3-21	4	22	3-20	2	21
	1.C02	CtxA/Holotoxin	1	IgG1	Igκ	3-21	4	28	3-20	2	17
	2.A05	CtxA/Holotoxin	1	IgG1	Igκ	3-21	4	30	3-20	2	22
	2.B02	CtxA/Holotoxin	1	IgG1	Igκ	3-21	4	34	3-20	2	23
	2.B11	CtxB	2	IgA2	Igλ	3-11	4	30	1-40	3	16
	2.C04	CtxB	2	IgG1	Igλ	3-11	4	31	1-40	3	20
	2.C10	CtxB	3	IgG1	Igλ	3-15	6	28	1-40	1	27
	1.C04	CtxB	4	IgG1	Igλ	3-11	4	31	1-40	3	18
	1.C12	CtxB	4	IgG1	Igλ	3-11	4	31	1-40	3	13
	1.A07	CtxB		IgG1	Igκ	5-51	4	18	3-20	2	16
	1.B04	CtxB		IgG2	Igλ	7-4	1	24	1-40	3	23
	2.C05	CtxB		IgG1	Igλ	4-39	5	28	2-23	1	15
	2.D05	CtxB		IgG1	Igλ	3-11	3	8	1-44	2	5
	2.B05	Sialidase		IgG1	Igκ	3-9	6	12	3-11	2	9
	1.C01	Undetermined		IgG2	Igλ	4-28	5	42	1-51	3	28
	1.D06	Undetermined		IgG1	Igλ	7-4	4	21	7-43	3	25
	2.A08	Undetermined		IgG2	Igκ	3-53	6	33	1-39	1	25
	2.B06	Undetermined		IgG1	Igλ	4-59	4	26	3-21	3	13
	2.C07	Undetermined		IgG1	Igλ	4-39	5	27	2-8	3	17
	2.D01	Undetermined		IgG1	Igκ	3-21	3	21	1-39	4	19
CF21	2.E01	CtxA/Holotoxin	4	IgA1	Igκ	3-21	3	16	4-1	1	9
	1.G01	CtxA/Holotoxin	4	IgG1	Igκ	3-21	3	23	4-1	1	17
	1.D03	CtxA/Holotoxin		IgG1	Igκ	4-31	4	27	2-30	1	10
	1.B02	CtxB		IgG1	Igκ	1-2	5	24	1-39	5	29
	2.B01	CtxB		IgG1	Igκ	4-59	4	28	1-39	3	19
	2.C02	CtxB		IgG1	Igλ	3-30	4	32	7-46	2	19

	1.A02	LPS/OSP	1	IgA2	Igκ	3-23	5	35	2-30	4	12
	1.A03	LPS/OSP	1	IgA2	Igκ	3-23	5	45	2-30	4	30
	1.A04	LPS/OSP	1	IgA2	Igκ	3-23	5	36	2-30	4	11
	2.F01	LPS/OSP	1	IgA2	Igκ	3-23	5	39	2-30	4	18
	1.B04	LPS/OSP	2	IgA1	Igκ	3-73	5	21	1-5	3	20
	1.C01	LPS/OSP	2	IgA2	Igκ	3-73	5	20	1-5	3	15
	1.D04	LPS/OSP	2	IgA2	Igκ	3-73	5	20	1-5	3	15
	1.F01	LPS/OSP	2	IgA1	Igκ	3-73	5	27	1-5	3	20
	1.D01	LPS/OSP	3	IgA1	Igλ	3-15	4	20	1-40	1	17
	2.G01	LPS/OSP	3	IgG1	Igλ	3-15	4	23	1-40	1	13
	1.C03	LPS/OSP		IgG2	Igκ	3-53	4	30	3-11	2	35
	1.B01	Undetermined	1	IgA2	Igκ	3-23	5	40	2-30	4	20
	1.F02	Undetermined	1	IgA2	Igκ	3-23	5	36	2-30	4	15
	1.E05	Undetermined		IgG2	Igκ	6-1	4	25	1-39	2	13
	1.A01	Undetermined		IgG1	Igκ	1-69	6	22	4-1	1	12
	2.D01	Undetermined		IgG1	Igκ	3-21	4	16	4-1	1	19
CF29	1.E03	CtxB	3	IgG1	Igκ	3-7	5	25	1-39	1	33
	1.E05	CtxB	3	IgG1	Igκ	3-7	5	15	1-39	1	31
	1.A05	CtxB	3	IgG1	Igκ	3-7	5	21	1-39	1	32
	1.A04	CtxB		IgG1	Igκ	3-23	3	25	1-5	1	20
	1.C01	CtxB		IgG1	Igκ	1-18	5	32	1-39	1	16
	1.A06	LPS/OSP	1	IgA2	Igκ	3-7	4	4	4-1	4	12
	1.B02	LPS/OSP	1	IgA2	Igκ	3-7	4	14	4-1	4	11
	1.B05	LPS/OSP	1	IgA1	Igκ	3-7	4	10	4-1	4	13
	1.C02	LPS/OSP	1	IgG2	Igκ	3-7	4	4	4-1	4	14
	1.G01	LPS/OSP	1	IgA1	Igκ	3-7	4	8	4-1	4	13
	1.G02	LPS/OSP	1	IgA1	Igκ	3-7	5	17	4-1	4	19
	1.E06	LPS/OSP	2	IgG1	Igκ	3-7	4	10	2-28	3	3
	1.A01	LPS/OSP	2	IgA1	Igκ	3-7	5	14	2-28	3	7
	1.A02	LPS/OSP	2	IgG1	Igκ	3-7	4	25	2-28	3	4
	1.C04	LPS/OSP	2	IgG1	Igκ	3-7	4	20	2-28	3	9
	1.G04	LPS/OSP	2	IgA1	Igκ	3-7	4	13	2-28	3	1
	1.G05	LPS/OSP	2	IgG1	Igκ	3-7	4	15	2-28	3	8
	1.C06	Sialidase		IgG1	Igκ	3-23	4	17	1-5	2	17
	1.B03	Undetermined		IgG1	Igλ	3-48	6	0	1-40	3	18
	1.B06	Undetermined		IgG1	Igλ	3-23	1	35	1-51	3	16
	1.C03	Undetermined		IgG1	Igκ	3-9	6	22	1-39	2	16
	1.D01	Undetermined		IgG1	Igκ	3-48	3	21	1-39	1	19
	1.F06	Undetermined		IgG1	Igκ	4-59	6	31	1-5	4	20
	1.G03	Undetermined		IgG1	Igκ	3-9	6	13	1-39	2	19
CF30	1.A03	CtxA/Holotoxin		IgG1	Igκ	3-11	6	17	4-1	1	13
	2.E02	CtxB	3	IgG1	Igκ	5-51	3	22	1-5	4	21
	1.E06	CtxB	3	IgG1	Igκ	5-51	3	22	1-5	4	26
	1.A05	Undetermined	1	IgA1	Igλ	5-51	5	31	2-14	2	28
	1.D04	Undetermined	1	IgA1	Igλ	5-51	5	31	2-14	2	28
	2.G02	Undetermined	2	IgA1	Igκ	3-30	4	16	4-1	1	12
	2.E05	Undetermined	4	IgM	Igκ	1-2	4	22	1-39	2	24
	2.B05	Undetermined	4	IgM	Igκ	1-2	4	25	1-39	2	15
	2.A03	Undetermined	5	IgM	Igκ	3-15	5	39	3-20	4	21
	2.G05	Undetermined	5	IgM	Igκ	3-15	5	19	3D-20	4	17
	1.A06	Undetermined	6	IgA1	Igκ	3-7	6	12	2-30	3	5
	2.C01	Undetermined	6	IgA1	Igκ	3-7	6	11	2-30	3	5
	1.E02	Undetermined		IgG1	Igκ	4-39	3	7	1-5	2	8
	1.E03	Undetermined		IgG1	Igκ	4-34	5	24	3-15	2	15
	1.A02	Undetermined		IgG1	Igκ	3-21	2	12	1-5	1	5
	1.C01	Undetermined		IgG1	Igλ	1-8	6	23	2-14	1	14
	1.C03	Undetermined		IgG2	Igκ	7-4	4	7	1-39	1	11
	1.C05	Undetermined		IgG2	Igλ	3-72	5	16	1-47	3	18
	2.A01	Undetermined		IgG2	Igκ	3-7	2	12	4-1	1	5
	2.A02	Undetermined		IgG1	Igλ	7-4	4	18	2-14	2	11

	2.B01	Undetermined		IgG1	Igκ	4-59	3	23	3-15	5	20
	2.B03	Undetermined		IgG1	Igκ	3-53	4	5	3-20	1	6
	2.C02	Undetermined		IgG1	Igλ	4-39	6	7	3-1	1	3
	2.G01	Undetermined		IgG3	Igκ	7-4-1	6	13	1-39	1	10
	2.G03	Undetermined		IgG1	Igλ	4-30	6	1	1-44	3	1
CF31	1.B05	CtxA/Holotoxin		IgG1	Igκ	3-21	4	20	3-20	2	20
	1.D02	CtxA/Holotoxin		IgG1	Igκ	3-21	6	20	4-1	2	13
	1.E02	CtxB	2	IgG1	Igκ	4-61	2	19	1-16	1	15
	1.C02	CtxB	2	IgG1	Igκ	4-61	2	30	1-16	1	23
	1.E04	CtxB	3	IgG1	Igκ	5-51	4	27	1D-12	2	20
	1.C05	CtxB	3	IgG1	Igκ	5-51	4	24	1-12	2	18
	1.E06	CtxB		IgG1	Igλ	3-30	6	24	3-21	1	26
	1.B06	CtxB		IgG1	Igλ	3-23	4	20	3-21	3	16
	1.F03	CtxB		IgG1	Igλ	3-30	6	19	3-21	1	28
	1.F05	CtxB		IgG1	Igλ	3-43	6	25	1D-33	5	13
	1.G01	CtxB		IgG1	Igλ	3-15	5	14	1-40	2	10
	1.G03	CtxB		IgG1	Igλ	1-2	4	22	1-40	3	8
	1.B04	LPS/OSP	1	IgG1	Igκ	3-23	4	29	3-20	4	14
	1.F02	Undetermined	1	IgM	Igκ	3-23	4	27	3-20	4	16
	1.A01	Undetermined		IgG1	Igκ	3-23	2	20	3-11	4	8
	1.A03	Undetermined		IgG1	Igκ	3-23	4	23	1-5	1	14
	1.A04	Undetermined		IgG1	Igκ	1-18	6	28	4-1	3	15
	1.F04	Undetermined		IgG2	Igλ	4-4	3	14	1-44	3	13
	1.G02	Undetermined		IgG2	Igκ	7-4	4	24	4-1	1	19
	1.G05	Undetermined		IgG1	Igκ	3-33	4	24	1-5	1	15

* All mAbs were cloned into heavy chain expression vectors containing the IgG1 constant region

† FlaA (Flagellin protein), TagA (Lipoprotein ToxR activated gene), PPK (Phosphocarrier protein kinase)
